# Interaction of the cotranslational Hsp70 Ssb with ribosomal proteins and rRNA depends on its lid domain

**DOI:** 10.1038/ncomms13563

**Published:** 2016-11-24

**Authors:** Andrea Gumiero, Charlotte Conz, Genís Valentín Gesé, Ying Zhang, Felix Alexander Weyer, Karine Lapouge, Julia Kappes, Ulrike von Plehwe, Géza Schermann, Edith Fitzke, Tina Wölfle, Tamás Fischer, Sabine Rospert, Irmgard Sinning

**Affiliations:** 1 Heidelberg University Biochemistry Center (BZH), INF 328, D-69120 Heidelberg, Germany; 2 Institute of Biochemistry and Molecular Biology, ZBMZ, Faculty of Medicine, University of Freiburg, D-79104 Freiburg, Germany; 3 BIOSS Centre for Biological Signaling Studies, University of Freiburg, D-79104 Freiburg, Germany

## Abstract

Cotranslational chaperones assist in *de novo* folding of nascent polypeptides in all organisms. In yeast, the heterodimeric ribosome-associated complex (RAC) forms a unique chaperone triad with the Hsp70 homologue Ssb. We report the X-ray structure of full length Ssb in the ATP-bound open conformation at 2.6 Å resolution and identify a positively charged region in the α-helical lid domain (SBDα), which is present in all members of the Ssb-subfamily of Hsp70s. Mutational analysis demonstrates that this region is strictly required for ribosome binding. Crosslinking shows that Ssb binds close to the tunnel exit via contacts with both, ribosomal proteins and rRNA, and that specific contacts can be correlated with switching between the open (ATP-bound) and closed (ADP-bound) conformation. Taken together, our data reveal how Ssb dynamics on the ribosome allows for the efficient interaction with nascent chains upon RAC-mediated activation of ATP hydrolysis.

## Abstract (web summary)

In yeast, the heterodimeric ribosome-associated complex (RAC) acts in concert with the Hsp70 protein Ssb, forming a unique chaperone triad. Here the authors use structural and biochemical approaches to shed light on how translation and folding are coupled in eukaryotes.

## Graphical abstract

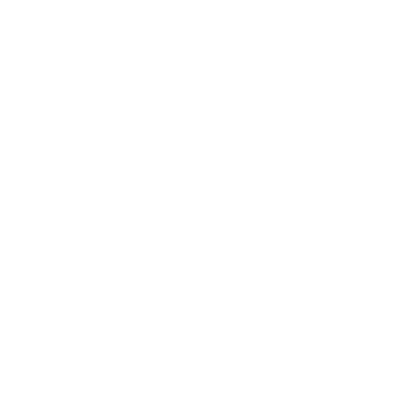

Already during translation at the ribosome, the emerging polypeptide chain is subject to enzymes for covalent modification, targeting factors for localization and chaperones for *de novo* protein folding^1^,^2^,^3^. In eukaryotes, the nascent polypeptide chain is modified by methionine amino-peptidases and N-acetyl transferases^2^,^4^,^5^. For targeting and insertion of membrane or secretory proteins into the endoplasmic reticulum membrane, the nascent chain is recognized and guided by the signal recognition particle (SRP) (refs 1, 6, 7, 8). To prevent aggregation and assist in folding of newly synthesized proteins, Hsp70 chaperones interact with nascent chains. Members of the Hsp70 family are highly conserved among all kingdoms of life and are known to bind short, largely hydrophobic sequences exposed by unfolded proteins^9^.

Hsp70 proteins share a number of structural and functional characteristics. They consist of a highly conserved N-terminal ATPase domain, also termed nucleotide binding domain (NBD), which is connected via a linker to the substrate binding domain (SBD). The SBD is divided into a β sandwich subdomain (SBDβ), an α-helical subdomain (SBDα or lid domain) and a less-conserved C-terminal domain of variable length^9^. The Hsp70–protein substrate interaction depends on ATP binding and on allosteric regulation between the NBD and the SBD. The ATP-bound state is characterized by a fast exchange rate of substrate (low affinity state), while in the ADP-bound state exchange is much slower (high affinity state)^10^. During the Hsp70 cycle, the chaperone switches between the ATP-bound state (open conformation) and the ADP-bound state (closed conformation) by major conformational rearrangements involving mainly the SBDα (refs 10, 11, 12, 13). Two types of cochaperones regulate the switch between the two states: Hsp40s stimulate ATP hydrolysis, and nucleotide exchange factors accelerate the exchange of ADP with ATP (refs 9, 14). Due to the high conservation, the allosteric mechanism recently established for DnaK likely applies to all canonical members of the Hsp70 family^15^.

Fungi from the large division of *Ascomycota* possess an evolutionary conserved subfamily of Hsp70s, termed the Ssb-type Hsp70s, which is named after two nearly identical homologues Ssb1 and Ssb2 (collectively Ssb) from *Saccharomyces cerevisiae*^16^. Ssb is distinguished from other Hsp70 homologues as it interacts directly with the ribosome by a mechanism that is currently unknown^3^,^17^. When bound to the ribosome, Ssb interacts with a large variety of nascent polypeptides and assists their cotranslational folding^18^. Because the ratio of ribosomes and Ssb in a yeast cell is near-balanced^19^ each ribosome could cooperate with one Ssb molecule throughout protein synthesis. However, only about 50% of Ssb is ribosome-associated at steady state, while the remainder is free in the cytosol^3^. This cytosolic pool of Ssb is involved in additional functions, not directly connected to cotranslational protein folding^3^,^20^,^21^.

Ribosome-bound Ssb cooperates with a specific cochaperone termed the ribosome-associated complex (RAC), which is composed of the Hsp70 protein Ssz1 and the Hsp40 protein Zuo1 (refs 22, 23, 24). Deletion of either *ZUO1*, *SSZ1* or *SSB1*/*SSB2* results in similar growth defects including slow growth, cold sensitivity and hypersensitivity towards paromomycin. The combined deletion of any of the above genes does not result in additive effects, suggesting that Ssb, Zuo1 and Ssz1 function together in the same pathway^3^,^25^,^26^. Of note the Ssb chaperone system is not induced at high temperature, but rather *SSB1* and *SSB2* are down-regulated together with ribosomal proteins on heat shock^3^,^27^. Rather than being heat shock proteins Ssb and RAC belong to the group of chaperones linked to protein synthesis^3^,^28^. The ribosome interaction of RAC has been characterized biochemically and structurally^22^,^29^,^30^,^31^. RAC binds close to the tunnel exit with contacts to Rpl22 and Rpl31 (refs 29, 30, 31), and several expansion segments of ribosomal RNA (rRNA), and stretches across the surface of the ribosome to the 40S ribosomal subunit^31^. Although the precise position of, for example the NBD of Ssz1 or the J-domain of Zuo1 are not resolved in the cryo-EM structures, the crosslink of Zuo1 to Rpl31 supports the idea that the J-domain is positioned close to the tunnel exit and possibly to Rpl31 (ref. 29). As the J-domain of Zuo1 activates the Ssb ATPase and switches it to the high affinity substrate binding state^32^, Ssb and the J-domain of Zuo1 should occupy neighbouring binding sites at the ribosome.

Here, we present a detailed structural and biochemical analysis of the Ssb–ribosome interaction. We determined the structure of full-length Ssb from the thermophilic *Ascomycota Chaetomium thermophilum*^33^,^34^ in the open ATP-bound conformation and characterized its ribosome binding by crosslinking and mutagenesis experiments. Ssb is positioned on the ribosome by dual interaction with ribosomal proteins and rRNA in close proximity of the tunnel exit. Ssb binding is modulated by RAC and our data allow to derive a model for the positioning and the dynamics of Ssb at the ribosome.

## Results

### Ssb interacts with ribosomal proteins at the tunnel exit

**Figure 1:**
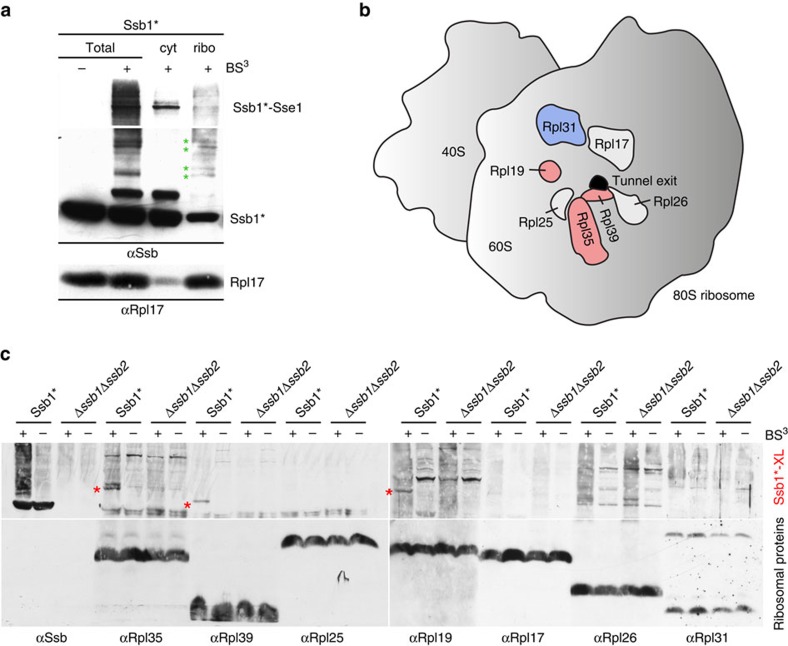
Ssb contacts ribosomal proteins at the tunnel exit. (**a**) Ssb is crosslinked to proteins of the large ribosomal subunit. Isolated ribosomes of the Ssb1* strain were incubated with (+) or without (−) the crosslinker BS^3^. The crosslinked sample (Total) was separated into a cytosolic supernatant (cyt) and a ribosomal pellet (ribo) via centrifugation through a low-salt sucrose cushion and was analysed via immunoblotting with αSsb. Shown is a short exposure of the upper part of the blot (>116 kDa), which contains the strong crosslink between Ssb1* and Sse1 (Ssb1*–Sse1) and a long exposure of the lower part of the blot (<116 kDa), which contains the weaker crosslinks between Ssb1* and ribosomal proteins (green asterisks). Rpl17 served as a marker for the ribosomal fraction. (**b**) Schematic representation of selected ribosomal proteins (Rpl19, Rpl35 and Rpl39 in pink; Rpl31 in blue; Rpl17, Rpl25 and Rpl26 in white) surrounding the tunnel exit (black) of the yeast ribosomal large subunit (grey). (**c**) Identification of ribosomal proteins crosslinked to Ssb1*. Crosslinking was performed in total cell extract of Ssb1* or Δ*ssb1*Δ*ssb2* strains. Aliquots were analysed via immunoblotting using αSsb, αRpl35, αRpl39, αRpl25, αRpl19, αRpl17, αRpl26 and αRpl31 as indicated. Crosslink products between Ssb1* and ribosomal proteins (Ssb1*-XL) are indicated with red asterisks.

To better characterize the interaction of Ssb with ribosomes, we first investigated its positioning via crosslinking experiments. To this end, we employed a Δ*ssb1*Δ*ssb2* strain expressing the point mutant Ssb1-A577K (termed Ssb1*), which displayed enhanced affinity for αSsb (Supplementary Fig. 1a). Ssb1* was ribosome associated and fully complemented growth of a Δ*ssb1*Δ*ssb2* strain (Supplementary Fig. 1b,c). Crosslinking in cell extract generated several crosslink products detected by αSsb (Fig. 1a). After separation of the cytosol from ribosomes via a sucrose cushion, a prominent crosslink of ∼160 kDa was detected in the cytosolic fraction, while additional crosslinks of weaker intensity were observed in the ribosomal fraction (Fig. 1a). The 160 kDa band represents a crosslink between Ssb1 and the cytosolic Sse1, which functions as a cochaperone of the cytosolic pool of Ssb (refs 35, 36, 37) (Fig. 1a; Supplementary Fig. 1d). Many of the weaker crosslinks migrated with a molecular mass compatible with crosslink products between Ssb and ribosomal proteins, most of which possess a molecular mass between 6 and 25 kDa. Consistently, most of the smaller crosslinks were recovered in the ribosomal pellet (Fig. 1a, green asterisks). We next probed the crosslinks obtained in a wild type or in a Δ*ssb1*Δ*ssb2* extract with antibodies directed against ribosomal proteins surrounding the tunnel exit (Fig. 1b). While no differences in the crosslinking pattern of Rpl25, Rpl17, Rpl26 or Rpl31 between the Ssb1* and Δ*ssb1*Δ*ssb*2 strains were observed, Rpl35 (13.9 kDa), Rpl39 (6.3 kDa) and Rpl19 (21.7 kDa) formed crosslink products of the expected molecular mass in the wild type but not in a Δ*ssb1*Δ*ssb2* extract (Fig. 1c). The general crosslinking pattern observed in extracts derived from wild type cells was similar, with bands of slightly weaker intensity, than observed in extracts from the Ssb1* strain (Supplementary Fig. 1e). The crosslink band detected with αRpl35 was fuzzy or, in some experiments, migrated as a doublet of bands (Fig. 1c), which both represented crosslinks between Ssb and Rpl35 (Supplementary Fig. 1f). From these data, we conclude that Ssb contacts Rpl35, Rpl39 and Rpl19, which are all located in close proximity of the tunnel exit.

### Structure determination employing a cysteine-crosslink

**Figure 2:**
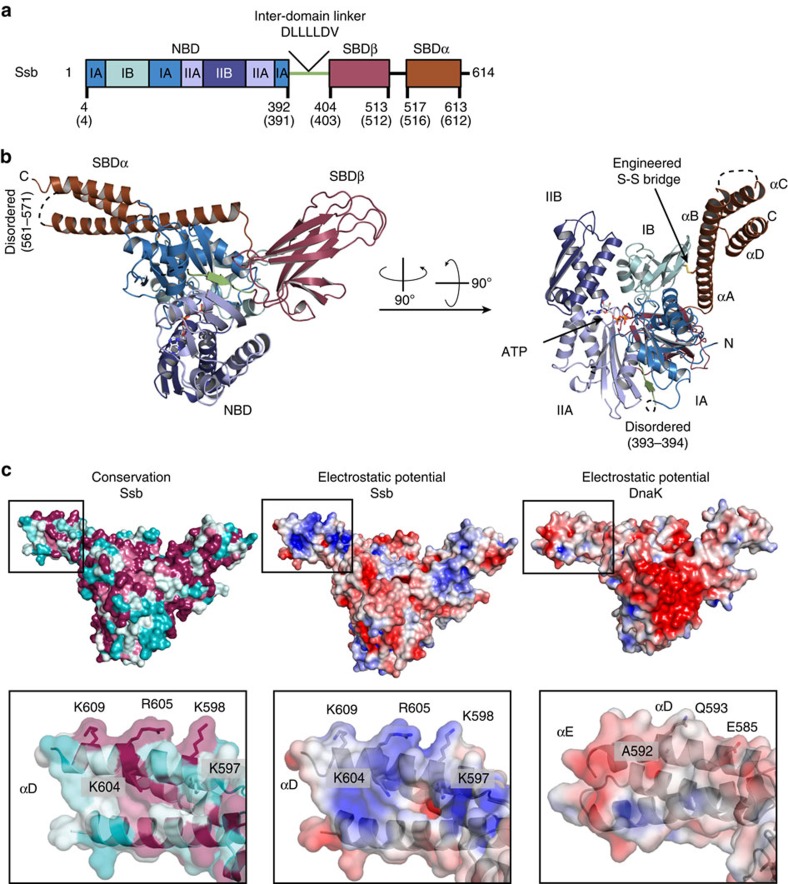
Crystal structure of Ssb. (**a**) Domain architecture of Ssb (residue numbers are given for *C. thermophilum;* corresponding residues in *S. cerevisiae* are in brackets). Ssb comprises an NBD (shades of blue) and an SBD consisting SBDβ (purple) and SBDα (lid domain, brown). A conserved linker (DLLLLDV, green) connects the NBD to the SBD. (**b**) Crystal structure of Ssb in the open conformation (cartoon representation). Ssb is a canonical Hsp70 with the NBD (subdomains IA, IIA, IB and IIB in different shades of blue) contacting both the SBDβ and the SBDα. SBDα is fixed on the NBD (bound ATP shown in sticks) by an engineered disulfide bridge (shown as sticks, see Supplementary Fig. 4). (**c**) Surface representation of Ssb (left and middle panel) and DnaK (right panel; pdb code 4b9q (ref. 11)) coloured by residue conservation (left panel) or electrostatic surface potential (middle and right panel). Insets show a close-up view of conserved residues from Ssb-SBDα forming a positively charged surface patch absent in DnaK. Surface charge spans from −4 kT/e (deep red) to +4 kT/e (deep blue). Conservation surface mapping are as reported by ConSurf^84^ and spans from variable (cyan) to conserved (deep red). Figures were produced using PDB2PQR (refs 71, 72) and APBS (ref. 70) in PyMOL (ref. 68).

Having identified the Ssb interacting ribosomal proteins, we wanted to understand the molecular details of the Ssb ribosome interactions. Therefore, we set out to determine the crystal structure of Ssb from the thermophilic fungus *Chaetomium thermophilum*^33^,^34^, which is 75% identical to Ssb1 from *Saccharomyces cerevisiae*. Within the Hsp70 family, domain organization is highly conserved (Fig. 2a; Supplementary Fig. 2; Ssb residue numbering is given for *C. thermophilum* unless stated otherwise). In order to crystallize full-length Ssb, we stabilized the ATP-bound, open conformation adapting a cysteine crosslinking strategy that was successfully used for the structure determination of *Escherichia coli* DnaK (ref. 11). To identify the relevant residues to create the cysteine bridge, we created a homology model of full-length Ssb based on DnaK (refs 11, 38) using SWISS-MODEL^39^,^40^. On the basis of this model, the conserved threonine in the NBD P-loop was replaced by an alanine (T208A) to reduce the intrinsic ATPase activity. Two cysteines (in the NBD and SBDα) had to be introduced to link both domains via a disulfide bond. While the position in the NBD was readily determined (E51), we could not directly identify the relevant position of the second cysteine from the homology model. Therefore seven residues (534–540) in Ssb SBDα were individually mutated. After purification and oxidation, only the Ssb T208A-E51C-D534C mutant crystallized in the presence of ATP (Supplementary Fig. 3a,b). The crystals belong to the monoclinic space group P 1 2_1_ 1 with unit cell parameters of *a*=66.9 Å, *b*=128.2 Å and *c*=79.7 Å (Table 1) and contain two molecules in the asymmetric unit. The structure was solved at 2.6 Å resolution by molecular replacement using DnaK as a search model^11^ (Table 1). The resulting electron density map was of high quality showing well defined ATP and Mg^2+^ ligands, as well as the engineered C51–C534 disulfide bridge (Supplementary Fig. 4). The final model has excellent stereochemistry (Ramachandran allowed 99.5%) and only small parts of the structure are disordered (residues 1–3, 393–394, 561–571 and 613–614), indicated as dotted lines (Fig. 2b).

### Crystal structure of ATP-bound Ssb

Ssb is a typical member of the Hsp70 family and the crystal structure shows the canonical domain architecture (NBD, SBDβ and SBDα) of Hsp70 chaperones (Fig. 2a,b). The Ssb nucleotide binding domain (NBD, blue) and the substrate binding domain (SBD, purple and brown) are connected by the conserved inter-domain linker DLLLLDV (green) responsible for allosteric communication^15^,^41^.

The Ssb NBD, the β-sheet and α-helical subdomains of the SBD are arranged as in the open (ATP-bound) conformation of DnaK (ref. 11). The NBD (residues 4–392) has the typical actin-like fold^42^ and comprises two lobes (I and II), further divided into four subdomains (IA, IB, IIA and IIB) (Fig. 2b). ATP binds between the two lobes. Aspartate 13 coordinates the Mg^2+^ ion through two water molecules, and Mg^2+^ in turn coordinates both the ATP β and γ phosphates (Supplementary Figs 4a and 5a). Superposition of the NBDs (including the active site) of Ssb with DnaK shows an r.m.s.d. below 1 Å (for 358 Cα atoms) underlining the high structural conservation (Supplementary Fig. 5b).

As in the ATP-bound open conformation of DnaK, the β-sheet subdomain of the SBD (Ssb-SBDβ, residues 404–513) contacts the NBD subdomains IA, IIA and IB (Fig. 2b). It shows the typical, distorted β-sandwich fold formed by two layers of β-sheets built by three and five strands (Supplementary Fig. 5c,d). In Hsp70s the residues involved in substrate binding are conserved and the loop between the first and second β-strand of the SBDβ (L_1–2_) is important for substrate binding as it contributes to the shape of the binding cleft (Supplementary Fig. 2). When we compare Ssb-SBDβ with DnaK-SBDβ two important differences are observed: methionine 410 (threonine 403 in DnaK) seems to restrict the entrance to the substrate binding cleft, and glutamate 411 (methionine 404 in DnaK) may be engaged in polar contacts with the substrate. Methionine 404 in DnaK forms van der Waals contacts with the central leucine of the substrate peptide^43^ (Supplementary Fig. 5c,d). These adaptations in Ssb-SBDβ are conserved in all members of the Ssb subfamily (Supplementary Fig. 2) but their influence on Ssb substrate specificity has not been addressed so far.

In Ssb, the SBDβ is followed by SBDα (residues 517–613, helical lid domain) that comprises four α-helices (A–D), with αB to αD forming a three-helix bundle. Our structure shows a high degree of flexibility for this helical bundle as indicated by high B-factors (Supplementary Fig. 6). In cytosolic Hsp70 proteins SBDα comprises five α-helices. In Ssb, the helix αC of Ssb-SBDα contains a nuclear export signal (NES) (ref. 44) (residues ^575^IEQALSEAM^583^) of which the hydrophobic side chains are engaged in van der Waals contacts forming the hydrophobic core of the bundle.

### The Ssb-SBDα is required for ribosome binding

**Figure 3:**
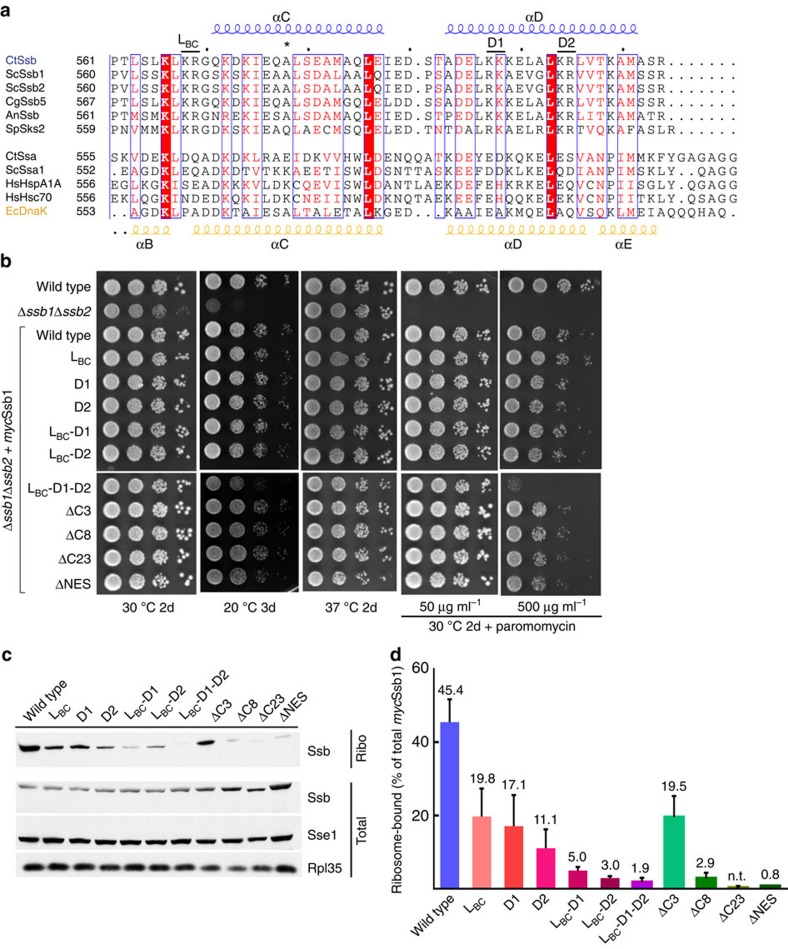
Ssb SBDα is required for ribosome-binding. (**a**) Sequence alignment of the SBDα C-terminal part from Ssb family members (upper block) and canonical Hsp70s (lower block). Secondary structure elements based on the crystal structure of CtSsb and EcDnaK are depicted on top in blue (helix αC and αD) and bottom in yellow (helix αB, αC, αD, and αE), respectively. Representation generated with ESPRIPT^74^. Ct: *Chaetomium thermophilum*, Sc: *Saccharomyces cerevisiae*, Cg: *Chaetomium globosum*, An: *Aspergillus nidulans*, Sp: *Schizosaccharomyces pombe*, Hs: *Homo sapiens*, Ec: *Escherichia coli*. *: indicates the position of the A577K mutation within Ssb1 (Ssb1*). The mutations within the positive patches are indicated above the CtSsb sequence (L_BC_, D1 and D2). (**b**) Mutants within the C-terminal 50 residues of Ssb1 rescued the growth defects of a Δ*ssb1*Δ*ssb2* strain. Serial 10-fold dilutions of strains expressing *myc*-tagged versions of Ssb1 were spotted onto YPD plates and were incubated as indicated. Paromomycin was added to plates as indicated. (**c**) Ssb ribosome-binding is affected by mutations in SBDα. Aliquots of cell extract (Total) and ribosomes isolated under low-salt conditions (Ribo) were analysed using α*myc*, αSse1 and αRpl35. Sse1 and Rpl35 in total extract are shown as loading controls. A longer exposure of the ribosome fraction blot is shown to visualize the Ssb variants with weak ribosome binding. (**d**) Quantitative analysis of the ribosome-bound fraction of Ssb variants. Quantifications are based on at least three independent experiments (Supplementary Fig. 7b); error bars represent the s.d. Ribosome-binding of the *myc*Ssb1-ΔC23 mutant was below the detection limit. Ssb L_BC_: K567E/R568E, Ssb D1: R596D/K597D and Ssb D2: K603D/R604D.

Further analysis of the Ssb structure in combination with a detailed sequence alignment revealed the presence of a positive patch located in SBDα. This positive patch is mainly formed by conserved Arg and Lys residues (K597, K598, K604, R605 and K609) located in the helices αC and αD (Fig. 2c, left and middle panel; Fig. 3a). These residues are conserved in members of the Ssb subfamily (Fig. 2c, left panel) but not in canonical Hsp70s, as shown by comparison with DnaK (ref. 11) (Fig. 2c, middle and right panel). To test whether these Ssb specific, positively charged residues are involved in Ssb–ribosome interaction, we created a series of mutants, in which the relevant positively charged residues had been replaced pairwise and performed quantitative ribosome binding assays (Fig. 3). The first pair K568/R569 (*Sc* K567/R568) is in a disordered loop connecting helices αB and αC (Ssb L_BC_), the second and third pair K597/K598 (*Sc* R596/K597) and K604/R605 (*Sc* K603/R604) are located in helix αD (Ssb D1 and Ssb D2, respectively). We created reverse charge double mutants in yeast Ssb1, resulting in three variants: Ssb L_BC_ (K567E/R568E), Ssb D1 (R596D/K597D) and Ssb D2 (K603D/R604D) (Fig. 3a). As the Ssb antibody is directed against the very C-terminus of Ssb, mutations within this region strongly affect antibody recognition (Supplementary Fig. 7a), therefore an N-terminally *myc*-tagged Ssb1 (*myc*Ssb1) was employed in these experiments (Supplementary Fig. 7a). All three mutants (L_BC_, D1, and D2) fully complemented growth defects of a Δ*ssb1*Δ*ssb2* strain (Fig. 3b). However, ribosome-binding of the three mutants was reduced to less than 50% of the *myc*Ssb1 control (Fig. 3c,d; Supplementary Fig. 7b). Due to possible synergistic effects of these mutations, double (L_BC_–D1, L_BC_–D2) and triple (L_BC_–D1–D2) mutants were tested. While double mutants fully complemented growth defects of the Δ*ssb1*Δ*ssb2* strain, the triple mutant displayed slightly reduced growth at 20 °C and at 30 °C in the presence of 50 μg ml^−1^ paromomycin (Fig. 3b). When the paromomycin concentration was raised to 500 μg ml^−1^, a concentration at which even growth of the wild type strain was reduced, the triple L_BC_–D1–D2 mutant displayed a severe growth defect (Fig. 3b). However, all three mutants were severely defective with respect to ribosome-binding (Fig. 3c,d). These findings indicated that the growth defect of the L_BC_–D1–D2 mutant was not due to a defect in ribosome binding, but was due to a negative effect caused probably by the accumulation of negative charges within the very C-terminal region of Ssb1. This is also supported by the observation that, consistent with previous data^16^, a series of Ssb1 C-terminal truncation variants, lacking the last three (ΔC3), eight (ΔC8) or twenty-three (ΔC23) residues fully supported growth of the Δ*ssb1*Δ*ssb2* strain (Fig. 3b), but displayed severe ribosome-binding deficiency (Fig. 3c,d; Supplementary Fig. 7b) as did internal deletions (ΔNES) (Fig. 3b–d; Supplementary Fig. 7b). In order to assay the effect of these variants on the integrity of the helical bundle, we analysed them by CD spectroscopy. As expected from the crystal structure, the ΔNES variant showed reduced helicity compared with the reverse charge mutants and the WT (Supplementary Fig. 3c,d). These effects could be explained by destabilization of the helical bundle due to disruption of the hydrophobic core. Taken together, our data indicate that the loop L_BC_ and helix αD as part of the three-helix bundle form an interaction platform that allows Ssb to efficiently bind to the ribosome. The interaction was strictly dependent on conserved, positively charged residues clustering into a three-dimensional epitope.

### Ssb–ribosome interaction is modulated by RAC

**Figure 4:**
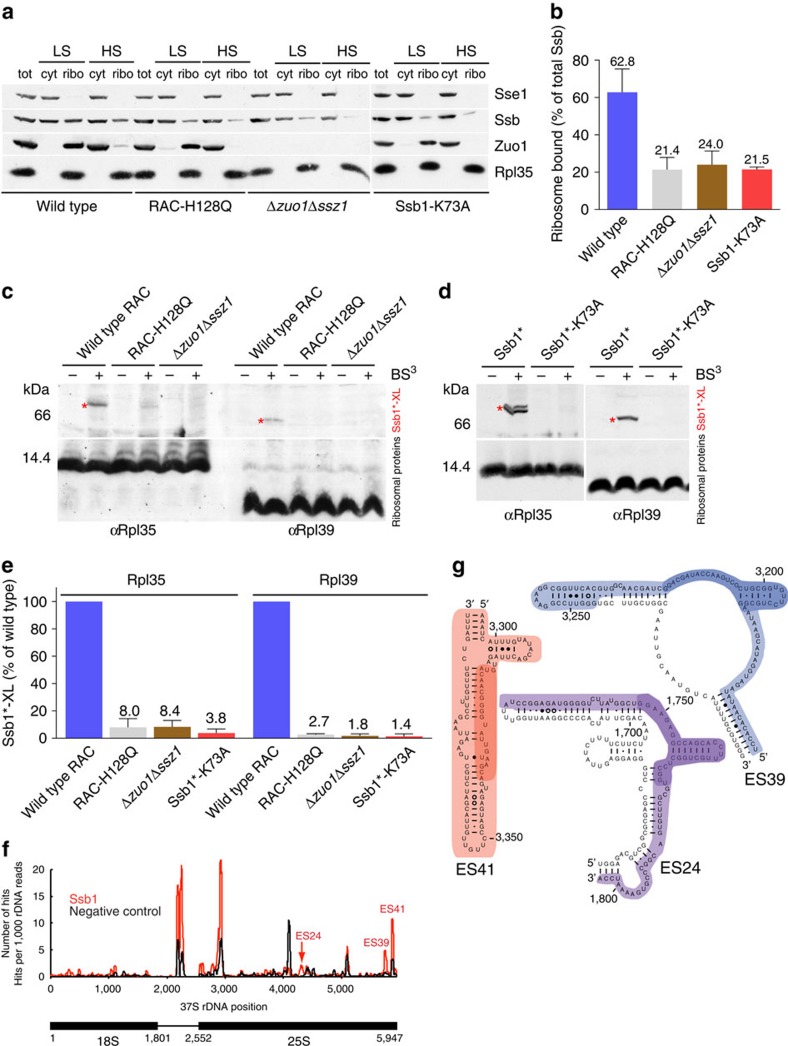
Ssb–ribosome interaction is modulated by RAC and involves rRNA expansion segments. (**a**) Ribosome-binding of Ssb is reduced when RAC is absent, non-functional, or if ATP hydrolysis is prevented by the Ssb1-K73A mutation. Total cell extract (tot) was separated into a cytosolic fraction (cyt) and a ribosomal pellet (ribo) under low-salt (LS) or high-salt (HS) conditions. Immunoblots were decorated with αSsb, αZuo1, αSse1 (cytosolic marker) or αRpl35 (ribosomal marker). (**b**) The ribosome-bound fraction of Ssb in extracts derived from RAC-H128Q, Δ*zuo1*Δ*ssz1* or Ssb1-K73A strains is reduced. Quantification of the ribosome-bound fraction of Ssb under low-salt conditions is based at least on 3 independent experiments (Supplementary Fig. 8), error bars represent the s.d. (**c**,**d**) The contact between Ssb1 and Rpl35 or Rpl39 is strongly reduced if RAC is absent, non-functional, or if ATP hydrolysis is prevented by the Ssb1-K73A mutation. Crosslinking was performed in cell extracts of strains expressing Ssb1* (Supplementary Table 1) and (**c**) wild type RAC, RAC-H128Q, or carrying the Δ*zuo1*Δ*ssz1* mutation or (**d**) carrying the Ssb1*-K73A mutation. Immunoblots were decorated with antibodies directed against Rpl35 or Rpl39. Crosslink products between Ssb1* and ribosomal proteins (Ssb1*-XL) are indicated with red asterisks. (**e**) Comparison of crosslinking efficiencies between Ssb1* and Rpl35 or Rpl39 in cell extracts derived from Ssb1* strains expressing wild type RAC, RAC-H128Q, carrying the Δ*zuo1*Δ*ssz1* mutation, or expressing Ssb1*-K73A. The band intensities of crosslink products between Ssb1* and Rpl35 (Ssb1*-Rpl35) or Rpl39 (Ssb1*-Rpl39) were determined in at least 3 independent experiments. The intensity of Ssb1*-Rpl35 and Ssb1*-Rpl39 crosslinks in the Ssb1* strain was set to 100%. Error bars represent the s.d. (**f**) CRAC analysis on ProtA-TEV-His_6_-Ssb1 (red) and negative control (black). The number of hits representing the number of times a nucleotide was mapped onto the 37S rDNA reference sequence of *S. cerevisiae* is shown. (**g**) Schematic representation of the expansion segments ES41, ES24 and ES39 of the 25S rRNA crosslinked to Ssb1 (ref. 85). Nucleotides found in all hits are highlighted and nucleotides representing the nucleotides frequently mutated or deleted in the CRAC experiments are highlighted in dark colour.

Ssb1 interacts with the ribosome by contacting Rpl35 and Rpl39 in close proximity to the tunnel exit. The Ssb cochaperone RAC (refs 3, 14) contacts Rpl31 and stimulates ATP hydrolysis in Ssb (Fig. 1b, blue)^29^,^30^,^31^,^32^. Therefore, we wanted to test whether RAC influences Ssb binding to the ribosome. We employed a strain lacking RAC (Δ*zuo1*Δ*ssz1*) or a strain where wild type Zuo1 was replaced by the Zuo1-H128Q variant (RAC-H128Q), which does not stimulate the ATPase activity of Ssb due to a mutation in the conserved HPD motif in the J-domain^32^. With respect to ribosome binding, RAC-H128Q behaves similar to wild type RAC, which fully binds to ribosomes under low-salt conditions and is released from ribosomes under high-salt conditions (Fig. 4a). However, ribosome binding of wild type Ssb1 was significantly reduced in a Δ*zuo1*Δ*ssz1* or in a RAC-H128Q strain (Fig. 4a,b; Supplementary Fig. 8) indicating that ribosome-association of Ssb was hampered when RAC was absent or non-functional as a cochaperone. We next probed the interaction of Ssb with Rpl35 and Rpl39 in the Δ*zuo1*Δ*ssz1* and RAC-H128Q strains employing the strong crosslinks of Ssb1 to Rpl35 and Rpl39 in the wild type strain (Figs 1c and 4c). The crosslink between Ssb1 and Rpl35 was reduced to less than 10% and the crosslink between Ssb1 and Rpl39 was reduced to less than 5% when RAC was absent or non-functional (Fig. 4c–e; Supplementary Fig. 8). These results suggested that RAC-stimulated ATP hydrolysis in Ssb, introducing the ADP-bound (closed) conformation, modulated the interaction of Ssb with Rpl35 and Rpl39. We therefore employed the Ssb1-K73A mutant, which is unable to hydrolyze ATP and thus does not complement growth defects of a Δ*ssb1*Δ*ssb2* strain^23^. Ssb1-K73A also showed moderately reduced binding to ribosomes, similar to the ribosome-binding defect observed in the absence of functional RAC (Fig. 4a,b). Importantly, however, crosslinking of Ssb1-K73A to Rpl35 and Rpl39 was severely reduced (Fig. 4d,e). Thus, the combined data strongly support a model in that in the closed conformation Ssb was in close contact to Rpl35 and Rpl39, while this was not the case in the open conformation.

### Ssb contacts specific expansion segments of rRNA

As the interaction of Ssb with nontranslating ribosomes is salt sensitive^3^,^18^ (Fig. 4a) and we detected a strict requirement of a positive surface patch for ribosome binding, we anticipated that ribosome binding of Ssb might involve interaction with ribosomal RNA. To experimentally test this hypothesis, we used the CRAC (ultraviolet crosslinking and analysis of complementary DNA (cDNA)) methodology^45^, which was previously used successfully to identify RNA-protein interactions in ribosomal subunits^45^,^46^,^47^. We found that Ssb1 directly contacts rRNA elements on the 60S subunit. Ssb1 was efficiently crosslinked to the eukaryote specific expansion segments ES24 and ES41 close to the tunnel exit and to ES39 more distant from the tunnel exit (Fig. 4f,g). These data support the contribution of the positively charged patch on Ssb to the interaction with rRNA.

## Discussion

Ssb interacts with nascent polypeptide chains when they emerge from the ribosomal tunnel exit^3^,^18^,^26^ and so it was long anticipated that Ssb must interact with the ribosome close to the polypeptide tunnel exit. However, the exact localization, molecular details of this interaction, and the interplay with its cochaperone RAC remained unclear.

The crystal structure in the open ATP-bound conformation shows that Ssb represents a canonical member of the Hsp70 family. Overall, the structure is highly similar to the *E. coli* Hsp70 homologue DnaK in its open conformation^11^,^48^. The structure was readily obtained using a crosslinking approach developed for DnaK (ref. 11) underlining the high conservation within the Hsp70 family. The nucleotide and substrate binding domains are connected by a conserved, hydrophobic linker region. The substrate binding site is more narrow compared with DnaK due to specific changes in SBDβ, probably reflecting different substrate specificity. The Ssb SBDα comprises four helices and displays high flexibility. All elements required for ATP binding, for substrate binding and for allosteric regulation are present, suggesting that Ssb follows the same molecular mechanism of allostery as DnaK (ref. 15).

Ssb SBDα exposes a positively charged surface region conserved in all members of the Ssb subfamily of Hsp70s, but not in canonical cytosolic Hsp70s such as Ssa. Comparison with the closed conformation of DnaK shows that also in the closed conformation of Ssb this region would be solvent exposed and contacts neither SBDβ nor the NBD, indicating that it is available to interact with another partner. To address if the positively charged patch is involved in the interaction of Ssb with ribosomes, we performed crosslinking experiments in combination with ribosome binding assays. A combination of structure based *in vivo* and *in vitro* mutational analysis of three positive patches within the SBDα lid domain demonstrated the importance of these residues for ribosome binding. Although these mutations impaired, or when combined, abolished ribosome binding of Ssb almost entirely, cell growth was not significantly affected. This observation suggests that the direct interaction of Ssb with ribosomes is less important than previously anticipated. However, the finding is not too surprising if one considers that Ssb-type Hsp70s are confined to *Ascomycota* and, for example, mammalian cells do not contain Ssb-type Hsp70 homologues, which directly interact with ribosomes^49^. Likely, when Ssb is not prepositioned on the ribosome, Ssa, the other major cytosolic Hsp70 in yeast, contributes to cotranslational protein folding and thereby compensates for the ribosome binding defects of the Ssb lid domain mutants^28^. Indeed, Ssa, together with the J-domain chaperone Jjj1 can assist cotranslational protein folding in yeast^50^.

**Figure 5:**
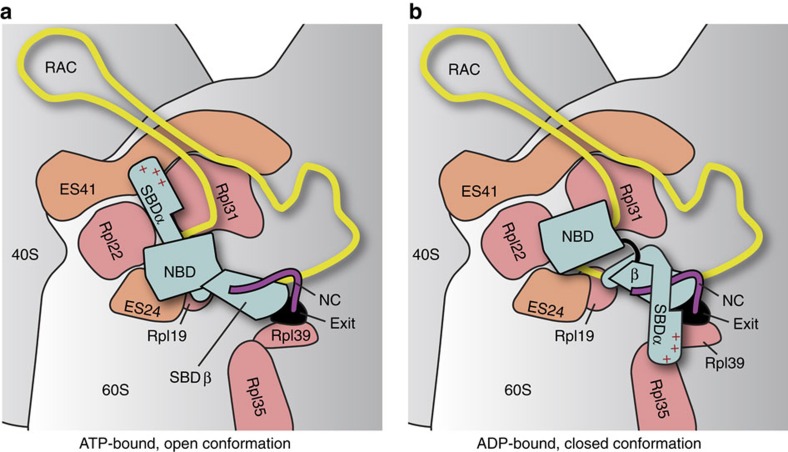
Model of Ssb binding at the ribosomal tunnel exit. (**a**) Ssb in the open conformation (ATP-bound, pre-hydrolysis state) binds close to the tunnel exit. The model is based on a molecular model of Ssb at the ribosomal tunnel exit (shown in Supplementary Fig. 9). In this state, the substrate (nascent chain, NC; purple) is not tightly bound (low affinity state). (**b**) On interaction with the cochaperone ribosome-associated complex (RAC), ATP is hydrolyzed and Ssb switches to the closed conformation (ADP-bound, post-hydrolysis state), which involves flipping of SBDα onto the SBDβ. Now Ssb can tightly interact with the nascent chain via SBD (high affinity state). The ribosome is shown in grey, the 60 S and 40 S subunits are indicated. Exposed expansion segments of rRNA (ES41, ES24) and ribosomal proteins (Rpl19, Rpl22, Rpl31, Rpl35, Rpl39) are depicted in orange and salmon, respectively. The tunnel exit is highlighted by a black circle (exit). Ssb is shown in blue and domains are labelled (NBD, SBDα, SBDβ).

Using CRAC methodology we identified three rRNA expansion segments (ES24, ES39 and ES41) on the 60S ribosomal subunit as direct interaction partners of Ssb. While ES24 and ES41 are located close to the tunnel exit (Fig. 5), ES39 is more distant. In the presence of RAC, ES39 appears shielded while ES41 and ES24 seem available for Ssb binding^30^,^31^. The crosslink to ES39 could therefore indicate an additional rRNA binding site of Ssb when RAC is absent. Using ribosome-binding experiments we also detected residue-specific contacts between SBDα and the ribosome. Our data indicate that Ssb interacts with Rpl35, Rpl39 and Rpl19 close to the tunnel exit. Using the crosslink data as restraints, we positioned the structure of ATP-bound Ssb on the ribosome^51^. SBDβ was docked close to the exit tunnel to allow for the interaction with a nascent chain and SBDα was oriented towards ES41. Choosing this orientation, SBDα is wedged in between Rpl22 and Rpl31, and within crosslinking distance to ES24 (Fig. 5a, Supplementary Fig. 9). While this orientation of the ATP-bound Ssb in the open conformation correlates with the crosslinks to ES41 and ES24, SBDα is not in close proximity of Rpl35 and Rpl39. We therefore created a model of Ssb in the closed conformation (based on DnaK (ref. 43)) as switching between the open and closed states relocates SBDα by >60 Å (Fig. 5b). When we docked a model of ADP-bound Ssb (closed conformation) via the SBDβ to the tunnel exit as before, SBDα is in close proximity of Rpl35 and Rpl39. Thus, docking Ssb to the ribosome in the open or closed conformation faithfully correlates with the observed protein and rRNA crosslinks and indicates that the structural rearrangements underlying allosteric regulation of Hsp70 proteins should also occur in Ssb and give rise to specific crosslinks. This model is further supported by the observation that the crosslinks to Rpl35 and Rpl39 were significantly reduced when Ssb cannot hydrolyze ATP (K73A mutant), as well as when RAC was absent or non-functional. Because RAC is strictly required for efficient ATP hydrolysis in Ssb (ref. 32), these data indicate that crosslinking to Rpl35 and Rpl39 was indeed confined to the closed (ADP-bound) conformation of Ssb. Consistently, it was previously shown that Ssb fails to interact with nascent chains in the absence of functional RAC (ref. 26), as the high affinity substrate binding state of Ssb is not induced. In summary, Ssb interacts with the 60S ribosomal subunit in a dual manner: by specific protein-protein as well as protein-rRNA contacts. Both types of interaction require the Ssb lid domain and are modulated by the cochaperone RAC.

The ribosomal surface around the tunnel exit, which serves as a binding platform for RAC and Ssb, has been described as an universal adaptor site for cotranslational factors including chaperones, enzymes and targeting factors^2^,^52^,^53^. It provides two main binding sites: Rpl25/Rpl35 (L23/L29 in *E. coli*) for SRP, trigger factor, the translocon, YidC/Oxa1 and Map and Rpl31/Rpl17 (L17/L22 in eubacteria) for RAC, the nascent chain associated complex (NAC), the SRP receptor and peptide deformylase. The crosslinks of Ssb to ES41, which is next to the Rpl31/Rpl17 site and to Rpl35, which is part of the other site, indicate that Ssb bridges both binding sites. Interestingly, during ribosome biogenesis control mechanisms are implemented to ensure that the universal adaptor site is not only correctly assembled, but also protected from premature, unproductive interactions^54^. The ribosomal surface around the tunnel exit directs the highly dynamic interplay of a myriad of factors and we only begin to understand these complex interaction networks.

## Methods

### Strains and plasmids

Strains and plasmids are listed in Supplementary Table 1 and Supplementary Table 2. The nomenclature for ribosomal proteins used in this study is provided in Supplementary Table 3.

DNA cloning and plasmid preparation were performed according to standard methods^55^. All constructs for the expression of mutants and tagged proteins were verified by sequencing and Western blot analysis.

The *Chaetomium thermophilum* (*Ct*) *SSB* coding sequence fused to an N-terminal His_6_ tag was PCR amplified from the *Ct* cDNA and cloned into pET24. The mutations T208A, E51C and D534C were introduced by site-directed mutagenesis as described by the manufacturer (QuikChange Lightning, Agilent Technologies). The coding sequence of the 3-helical bundle of Ssb (residues 536–614) fused to an N-terminal His_6_ tag was amplified by PCR from pET24a-His_6_Ssb-E51C-T208A-D534C and cloned into the pET24a vector. Mutations L_BC_ (K568E/R569E), D1 (K597D/K598D), D2 (K604D/R605D), ΔNES (Δ575-587) or a combination of them were introduced by site-directed mutagenesis.

The *Saccharomyces cerevisiae* (*Sc*) strains MH272-3f and BY4742 are the parental wild type strains of all haploid strains used in this study. MH272-3f strains lacking *SSB1* and *SSB2* (Δ*ssb1*Δ*ssb2*), *ZUO1 (*Δ*zuo1*), *SSZ1 (*Δ*ssz1*) or lacking combinations of these genes were described previously^22^,^23^,^56^. The strain lacking *SSB1*, *SSB2* and *ZUO1 (*Δ*ssb1*Δ*ssb2*Δ*zuo1*) was generated by mating Δ*zuo1*Δ*ssz1* (ref. 22) with Δ*ssb1*Δ*ssb2* (ref. 23) followed by dissection and tetrade analysis. The *SSB1* open reading frame +/−300 bp up- and down-stream was cloned into pYCPlac33 (ref. 57). The resulting plasmid pYCPlac33-Ssb1 was used as a template to generate a single alanine to lysine exchange at position 577 of Ssb1 via QuikChange resulting in the low copy plasmid pYCPlac33-Ssb1*. SSB1* ±300 bp was transferred into the high copy plasmid pYCPlac195 resulting into pYCPlac195-Ssb1* (ref. 57). The Ssb1-K73A mutant contains an amino acid exchange within the ATPase domain of Ssb1, which prevents ATP hydrolysis^23^,^58^. To generate Ssb1*-K73A, the PstI/BglII fragment in pYCPlac33-Ssb1-K73A was replaced by the PstI/BglII from pYCPlac33-Ssb1-A577K. To obtain N-terminally *myc*-tagged (MEQKLISEEDL) versions of Ssb1, the *SSB1* open reading frame was cloned into pCM190 (Euroscarf) resulting in pCM190-*myc*Ssb1. Mutations within the Ssb1 SBDα were introduced by PCR using pCM190-*myc*Ssb1 as a template. Deletion of residues 574–586 within Ssb1 resulted in pCM190-*myc*Ssb1-ΔNES. Deletion of the C-terminal residues SSR (ΔC3), VTKAMSSR (ΔC8) and SADELRKAEVGLKRVVTKAMSSR (ΔC23) resulted in pCM190-*myc*Ssb1-ΔC3, pCM190-*myc*Ssb1-ΔC8 and pCM190-*myc*Ssb-ΔC23, respectively. All pCM190-derived *myc*Ssb expression plasmids were transformed into the Δ*ssb1*Δ*ssb2* strain. Zuo1-H128Q (ref. 26) was subcloned into pYCPlac181 (ref. 57) resulting in pYCPlac181-Zuo1-H128Q.

For CRAC analysis, the integration of the ProtA-TEV-His_6_ tag at the N-terminus of the *SSB1* coding sequence into the BY4742 genome was performed as described^59^,^60^. In brief, a cassette containing the clonNAT resistance gene linked to a 390 nucleotides region upstream of *SSB1* coding sequence fused to the ProtA-TEV-His_6_ tag sequence was PCR amplified using forward S1 and reverse S4 primers^59^,^60^ and the purified PCR product was transformed into BY4742. The transformants were selected on YPD (1% yeast extract, 2% peptone and 2% dextrose) supplemented with 100 μg ml^−1^ clonNAT agar plates.

### Protein preparation and crystallization

The His_6_Ssb-E51C-T208A-D534C variant was produced in *E. coli* BL21(DE3) Rosetta2 cells. Lysogeny Broth (LB) media cultures supplemented with 1.75% (w/v) lactose, kanamycin (50 μg ml^−1^) and chloramphenicol (34 μg ml^−1^) were grown for 18 h at 30 °C. Cells were harvested and resuspended in lysis buffer (20 mM Hepes/NaOH pH 8, 250 mM NaCl, 20 mM MgCl_2_, 20 mM KCl), lysed in a microfluidizer (M1-10 L, Microfluidics) and purified by IMAC (Immobilized metal ion affinity chromatography, HisTrap HP 1 ml, GE Healthcare): after application of the lysate, the column was washed with 20 column volumes of lysis buffer supplemented with 20 mM imidazole and was eluted with 5 column volumes of lysis buffer supplemented with 200 mM imidazole. The eluate was diluted 10 fold with ATP binding buffer (20 mM Hepes/NaOH pH 7.5, 20 mM NaCl, 5 mM MgCl_2_, 0.1 mM EGTA (ethylene glycol-bis(β-aminoether)-N,N,N′,N′-tetraacetic acid), 1 mM DTT (1,4-Dithiothreitol), 10% (v/v) glycerol) and was applied to an agarose column with immobilized N6-(6-Amino)hexyl-ATP (Jena Biosciences). The column was washed with 20 column volumes of ATP binding buffer supplemented with 500 mM NaCl and eluted with 10 column volumes of ATP binding buffer supplemented with 5 mM ATP. The eluate was diluted 10-fold with ATP binding buffer containing 5 mM ATP but lacking DTT and concentrated 10-fold using centrifugal filter units (Merck Millipore).

The oxidation of the disulfide bond (C51–C534) was catalysed by the addition of a CuSO_4_ and 1,10-Phenanthroline solution with a final concentration of 0.5 mM and 1.75 mM, respectively, and incubation for 30 min at room temperature. To minimize the formation of inter-molecular disulfide bonds, the concentration of the protein was kept below 10 μM. The oxidized Ssb was further purified by size exclusion chromatography (SEC; S200-26/60, GE Healthcare) in SEC buffer (20 mM Hepes/NaOH pH 7.5, 150 mM NaCl, 5 mM MgCl_2_, 10 mM KCl, 10% (v/v) glycerol).

Crystallization screens were performed at 291 K by the sitting-drop vapor-diffusion method upon mixing equal volumes (0.2 μl) of the Ssb protein solution (10 mg ml^−1^) and reservoir solution containing 20% (v/v) PEG 3350 and 0.2 M NH_4_H_2_PO_4_. Crystals grew after 3 days.

The three-helix bundle of Ssb and variants were expressed in *E. coli* BL21(DE3) Rosetta2 cells grown in LB media supplemented with kanamycin and chloramphenicol. Overexpression was induced with isopropyl-1-thio-β-D-galactopyranoside (IPTG) at an OD_600_ of 0.8–1.0 and the culture incubated overnight at 18 °C. Cells were harvested and resuspended in lysis buffer (20 mM Hepes/NaOH pH 7.5, 300 mM NaCl, 20 mM MgCl_2_, 20 mM KCl), lysed in a microfluidizer and purified by IMAC: after application of the lysate, the column was washed with 20 column volumes of lysis buffer supplemented with 20 mM imidazole and eluted with 5 column volumes of lysis buffer supplemented with 200 mM imidazole. The eluate was applied to a SEC column S200 26/60 (size exclusion chromatography, GE Healthcare) run in SEC buffer (20 mM Hepes/NaOH pH 7.5, 300 mM NaCl, 5 mM MgCl_2_, 10 mM KCl). Samples were dialyzed overnight at 4 °C against CD buffer (10 mM KH_2_PO_4_/K_2_HPO_4_ pH 7.5, 150 mM KF).

### Data collection and structure determination

Crystals were flash-frozen in liquid nitrogen after cryo-protection by transfer into cryo-solution containing mother liquor and 20% (v/v) glycerol. Diffraction data were measured under cryogenic conditions (100 K; Oxford Cryosystems Cryostream) at the European Synchrotron Radiation Facility (ESRF, Grenoble). Data were processed with XDS (ref. 61). Phases were obtained by molecular replacement using PHASER (ref. 62) (search model 4b9q^11^) in the PHENIX software package^63^. Model building, refinement and validation were done using COOT (ref. 64), *Phenix.refine*^65^, REFMAC5 (ref. 66) and MOLPROBITY^67^, respectively. Figures were prepared in PyMOL (ref. 68) and UCSF-Chimera^69^. Electrostatic surface potentials were calculated with APBS and PDB2PQR (refs 70, 71, 72) integrated in PyMOL (ref. 68). Sequence alignments were performed using Clustal Omega^73^ and visualized with ESPript 3.0 (ref. 74) ( http://espript.ibcp.fr/ESPript/ESPript/). Protein sequence conservations were analysed using the ConSURF server^75^,^76^.

### Phenotypic assays

Growth defects were analysed by spotting 10-fold serial dilutions containing the same number of cells onto YPD or YPD+25, 50 or 500 μg ml^−1^ paromomycin agar plates, and were incubated for 2 days at 30 °C or 37 °C or 3 days at 20 °C, as indicated.

### CRAC analysis

*In vivo* CRAC experiments were performed using the BY4742 ProtA-TEV-His_6_-Ssb1 and the parental BY4742 strain as negative control. Yeast cultures were grown to an OD_600_ of 2.0 in YPD, harvested and suspended in PBS (phosphate-buffered saline) before ultraviolet-irradiation in a Megatron UV chamber (1.6 J cm^−2^) for 3 min. The RNA crosslinked to the protein of interest was treated as described^45^,^47^ with the omission of transfer to nitrocellulose. Bands corresponding to the size of the protein of interest and higher were excised directly from the Bis-Tris NuPAGE gel (4–12%, Novex) and were subsequently digested by proteinase K. Extracted RNAs were amplified by RT-PCR. The obtained cDNAs were sequenced using the Illumina HiSeq sequencing platform and the reads were treated and analysed as described^77^ with one modification: the reads were mapped to *S. cerevisiae* genomic reference sequence using Bowtie 2 (v 2.2.5) (ref. 78). Two independent experiments were performed for each sample and one representative experiment is shown.

### Crosslinking assays

Protein crosslinking reactions were performed with the homobifunctional, amino-reactive crosslinker BS^3^ (bis-(sulfosuccinimidyl)-suberate, spacer length 1.14 nm, Thermo Scientific) either with isolated ribosomes^29^ or with total cell extracts prepared from freshly harvested yeast cells, rapidly frozen in liquid nitrogen, and subsequently disrupted with a cryo-mill (MM400, Retsch)^79^. About 500 μl of the cyro-mill cell powder (stored at −80 °C) was then resuspended in 1,200 μl crosslinking buffer (20 mM Hepes/KOH pH 7.4, 80 mM K(H_3_CCOO), 2 mM Mg(H_3_CCOO)_2_, 1 mM PMSF (phenylmethylsulfonyl fluoride), 2 mM DTT), cell debris were removed by centrifugation at 20,000 *g* for 2 min at 4 °C, and aliquots of the supernatant were then incubated in the absence or in the presence of 1.2 mM BS^3^ for 20 min at 21 °C. Crosslinking reactions were quenched by the addition of glycyl-glycine to a final concentration of 30 mM. Ribosomes and crosslinks to ribosomal proteins were collected by centrifugation at 180,000 *g* for 35 min. Ribosomal pellets were resuspended in 100 μl ribosome-binding buffer and were precipitated by addition of 5% trichloroacetic acid (TCA). TCA pellets were analysed on 10% Tris-Tricine gels followed by immunoblotting.

### Ribosome binding assays

Yeast strains were grown to early log phase on YPD, cycloheximide was added to a final concentration of 100 μg ml^−1^, and subsequently cells were harvested via centrifugation at 4,500 *g*. Cell pellets were resuspended in ribosome-binding buffer (20 mM Hepes/KOH pH 7.4, 2 mM Mg(H_3_CCOO)_2_, 120 mM K(H_3_CCOO), 100 μg ml^−1^ cycloheximide, 2 mM DTT, 1 mM PMSF, protease inhibitor mix: 1.25 μg ml^−1^ leupeptin, 0.75 μg ml^−1^ antipain, 0.25 μg ml^−1^ chymostatin, 0.25 μg ml^−1^ elastinal, 5 μg ml^−1^ pepstatin A) and disrupted using the glass beads method^80^. After a clearing spin at 20,000 *g*, each 60 μl of the total glass beads extract (A_260_ between 40 and 250) was loaded onto a 90 μl low-salt sucrose cushion (25% (w/v) sucrose, 20 mM Hepes/KOH pH 7.4, 120 mM K(H_3_CCOO), 2 mM Mg(H_3_CCOO)_2_, 2 mM DTT, 1 mM PMSF, protease inhibitor mix) or onto a 90 μl high-salt sucrose cushion (25% (w/v) sucrose, 20 mM Hepes/KOH, pH 7.4, 600 mM K(H_3_CCOO), 2 mM Mg(H_3_CCOO)_2_, 2 mM DTT, 1 mM PMSF, protease inhibitor mix). After centrifugation at 400,000 *g* at 4 °C for 25 min the cytosolic supernatant was collected and the ribosomal pellet was resuspended in 100 μl ribosome-binding buffer. Aliquots of the total glass beads extract, cytosolic supernatant, and resuspended ribosomal pellets were precipitated by addition of 5% TCA. TCA pellets were analysed on 10% Tris-Tricine gels followed by immunoblotting. For quantification purposes, the loading of cytosolic supernatant and ribosomal pellet was adjusted such, that the intensities of both bands on immunoblots were in the linear range of the analysis (Supplementary methods). Quantification was performed using AIDA ImageAnalyzer (Raytest). For statistical analysis at least 3 independent experiments were examined.

### Circular dichroism spectroscopy

Sample concentration was measured by Bradford assay against a BSA standard (Rotiquant Bradford reagent, Roth) and adjusted to 20 μM. The CD spectroscopy measurements were performed at room temperature in a ultraviolet-spectropolarimeter J750 (Jasco) using 1 mm quartz cuvettes (Hellma).

### Immunoblotting

Proteins were separated on 10% Tris-Tricine gels. The following polyclonal antibodies were raised against peptides or purified proteins in rabbit: αSsb (ref. 19) (dilution 1:5,000), αRpl17 (ref. 19; dilution 1:20,000), αRpl24 (ref. 19; dilution 1:20,000) αRpl35 (dilution 1:20,000), αRpl39 (ref. 81; dilution 1:10,000), αRpl25 (ref. 82; dilution 1:10,000), αRpl19 (dilution 1:1,000), αRpl26 (dilution 1:4,000), αRpl31 (ref. 82; dilution 1:2,000), αSse1 (ref. 83; dilution 1:10,000) and αZuo1 (ref. 19; dilution 1:10,000) from EUROGENTEC (Bel S.A.). Monoclonal mouse αHis-tag (dilution 1:20,000, AbDSerotec, catalogue number: MCA1396) and α*myc*-tag (dilution 1:5,000, Chemicon, catalogue number: CBL430) antibodies were used as indicated. Immunoblots were developed using ECL with horse-radish peroxidase-conjugated goat α-rabbit IgG (dilution 1:10,000, Pierce, catalogue number: 31460) or α-mouse (dilution 1:5,000, Santa Cruz Biotechnology, catalogue number: Sc-2005) as secondary antibody. Uncropped blots are shown in Supplementary Figs 10–29. Validation of previously unpublished antibodies is shown in Supplementary Fig. 30.

### Data availability

Coordinates and structure factors have been deposited with the Protein Data Bank (PDB) under the accession codes: 5TKY. The data that support the findings of this study are available from the corresponding authors on request.

## Additional information

**How to cite this article**: Gumiero, A. *et al*. Interaction of the cotranslational Hsp70 Ssb with ribosomal proteins and rRNA depends on its lid domain. *Nat. Commun.* **7**, 13563 doi: 10.1038/ncomms13563 (2016).

**Publisher's note**: Springer Nature remains neutral with regard to jurisdictional claims in published maps and institutional affiliations.

## Supplementary Material

Supplementary Information Supplementary Figures 1 - 32, Supplementary Tables 1 - 3, Supplementary Methods and Supplementary References

## Figures and Tables

**Table 1:** Data collection and refinement statistics (molecular replacement).

	**CtSsb**
*Data collection*	
Space group	P 1 2_1_ 1
Cell dimensions	
a, b, c (Å)	66.9, 128.2, 79.7
α, β, γ (°)	90.0, 93.2, 90.0
Resolution (Å)	46.4–2.6 (2.69–2.60)*
R_merge_ (%)	10.3 (125.7)
*I/σI*	8.6 (0.9)
Completeness (%)	98.0 (99.0)
Redundancy	3.1 (3.2)
	
*Refinement*	
Resolution (Å)	46.4–2.6
No. of unique reflections	40,587 (4,085)
R_work_/R_free_(%)	22.8/26.4
No. of atoms	
Protein	9,123
Ligand/ion	64
Water	150
Average B-factors (Å^2^)	
Protein	59.1
Ligand/ion	42.6
Water	45.6
R.m.s. deviations	
Bond lengths (Å)	0.002
Bond angles (°)	0.55

r.m.s., root mean squared.

^*^Values in parentheses are for highest-resolution shell.
