# Supplementary material for: Interaction of the cotranslational Hsp70 Ssb with ribosomal proteins and rRNA depends on its lid domain
Source: Nat Commun. 2016 Nov 24;7:13563. doi: 10.1038/ncomms13563 (PMC5123055; doi:10.1038/ncomms13563)
Supplement: Supplementary Information — Supplementary Figures 1 - 32, Supplementary Tables 1 - 3, Supplementary Methods and Supplementary References [file ncomms13563-s1.pdf]

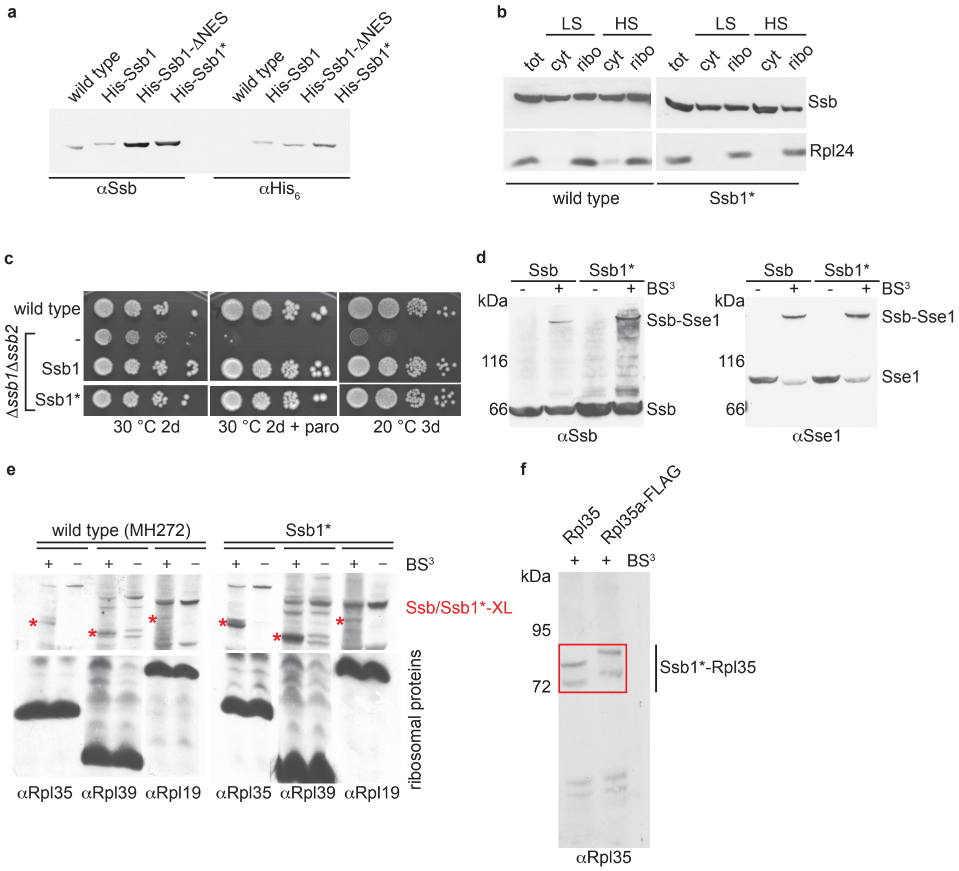

Supplementary Figure 1 │ Characterization of Ssb1-A577K (Ssb1*). (**a**) The A577K mutation within Ssb1 (Ssb1*) enhances recognition by αSsb. Aliquots of total protein extracts derived from galactose-grown wild type or Δ*ssb1*Δ*ssb2* strains expressing His_6-Ssb1, His_6-Ssb1-ΔNES, or His_6-Ssb1* (Supplementary methods) were analyzed via immunoblotting using αSsb or αHis_6. (**b**) Ribosome-binding of Ssb1* under low-salt (LS) and high-salt (HS) conditions analysed by immunoblotting using αSsb and αRpl24. (**c**) Complementation of the Δ*ssb1*Δ*ssb2* deletion by Ssb1*. Log-phase cells of wild type, Δ*ssb1*Δ*ssb2,* and Δ*ssb1*Δ*ssb2* strains complemented with plasmids encoding for Ssb1 or Ssb1* were spotted onto YPD plates and were incubated as indicated. The paromomycin concentration in plates (+ paro) was 25 µg ml^{-1}. (**d**) αSsb recognizes crosslinks to Ssb1* more efficiently than crosslinks to wild type Ssb. Total extracts of Δ*ssb1*Δ*ssb2* expressing either Ssb or Ssb1* were crosslinked with BS^3 (+ BS^3). As a control an aliquot was incubated without BS^3 (- BS^3). Samples were then split and were analyzed via immunoblotting using αSsb or αSse1. Ssb-Sse1 indicates the prominent crosslink product between Ssb/Ssb1* and Sse1. (**e**) The crosslinking pattern of wild type Ssb and Ssb1* is similar. Crosslinking was performed in total cell extracts of wild type or Ssb1* strains. Aliquots were analyzed via immunoblotting using antibodies directed against Rpl35, Rpl39, or Rpl19 as indicated. Crosslink products between Ssb/Ssb1* and ribosomal proteins (Ssb/Ssb1*-XL) are indicated with red asterisks. (**f**) The BS^3 crosslink between Ssb and Rpl35 runs as double band. Total extracts of Δ*ssb1*Δ*ssb2* expressing Ssb1* or Δ*ssb1*Δ*ssb2*Δ*rpl35a*Δ*rpl35b* expressing Ssb1* and Rpl35a-FLAG (Supplementary methods) were crosslinked with BS^3. Samples were analyzed via immunoblotting using αRpl35. The crosslinks between Ssb1* and Rpl35a or Rpl35a-FLAG, respectively, are boxed in red.

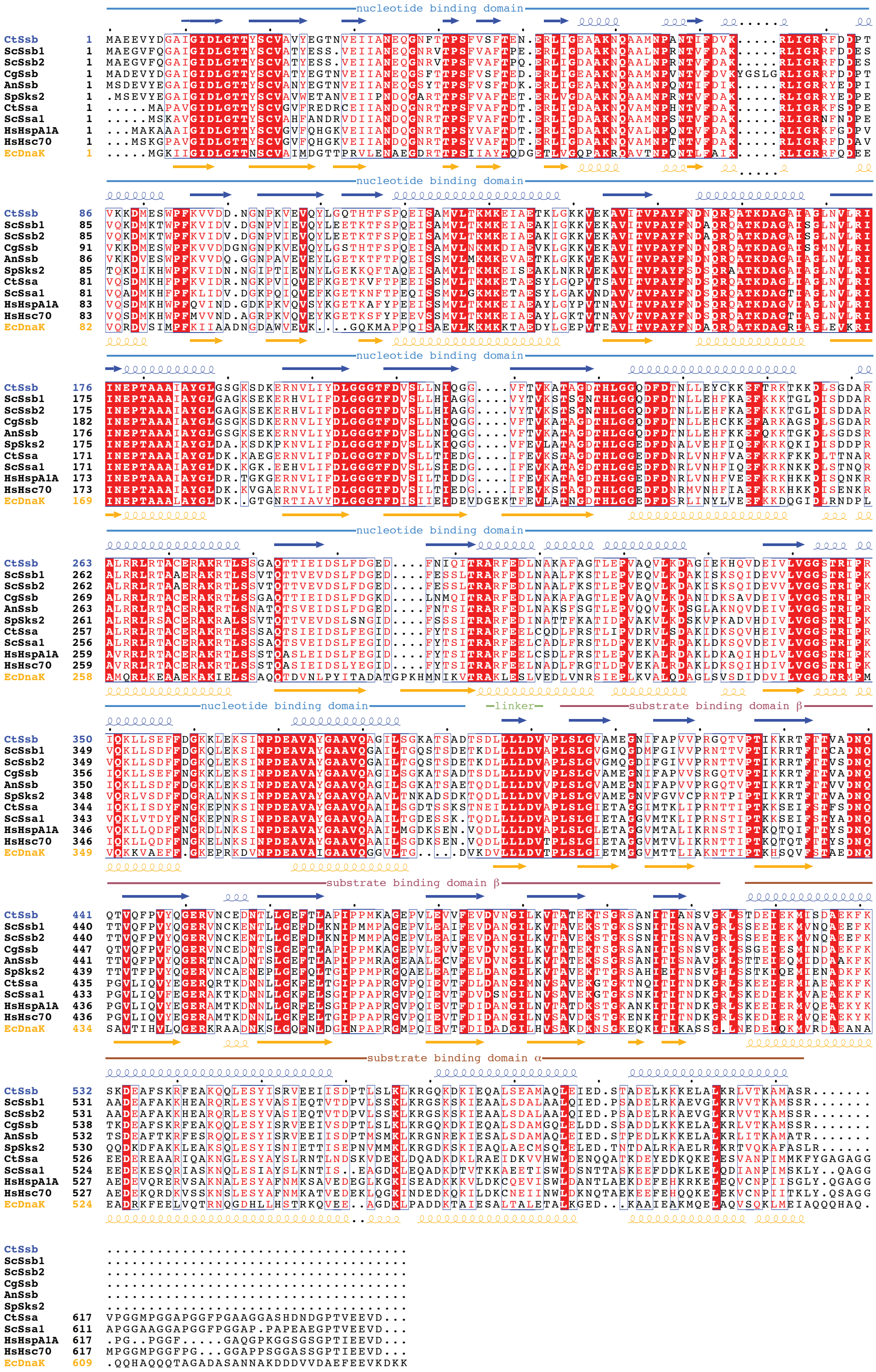

Supplementary Figure 2 | Multiple sequence alignment of Ssb family members and cytosolic Hsp70s. Domain boundaries and secondary structure elements based on the crystal structure of CtSsb are depicted on the top. Ct: *Chaetomium thermophilum*, Sc: *Saccharomyces cerevisiae*, Cg: *Chaetomium globosum*, An: *Aspergillus nidulans*, Sp: *Schizosaccharomyces pombe*, Hs: *Homo sapiens*, Ec: *Escherichia coli*.

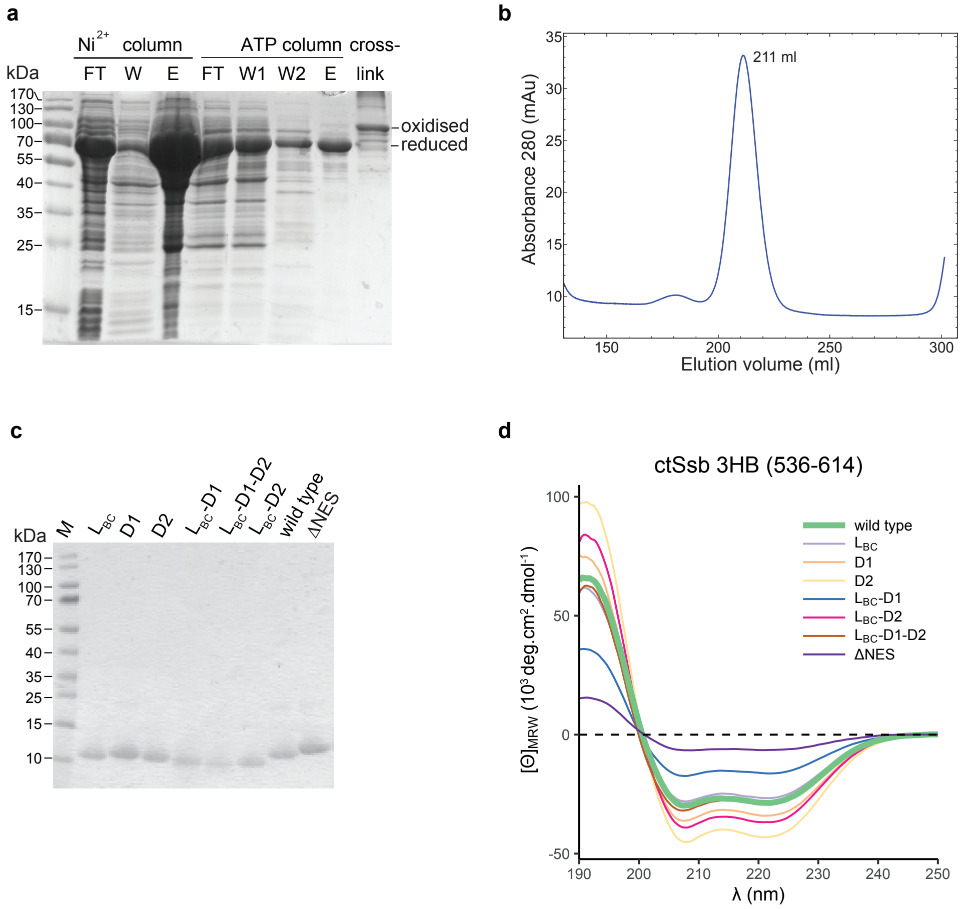

Supplementary Figure 3 | Purification of Ssb for crystallization and CD spectroscopy. (**a**) IMAC (Ni^{2+} column) and ATP affinity chromatography (ATP column) of Ssb E51C-T208A-D534C. FT: flow-through, W: wash, E: elution, W1: first wash with lysis buffer supplemented with 20 mM imidazole, W2: second wash with lysis buffer supplemented with 500 mM NaCl. The last lane depicts the Ssb sample under non-reducing condition (oxidized form). (**b**) Size exclusion chromatography profile of oxidized Ssb. (**c**) SDS-PAGE of the purified Ssb 3HB (536-614) wild type and mutants used for the CD spectroscopy experiments. (**d**) CD spectrum showing the mean residue ellipticity of the C-terminal 3-helix bundle of Ssb.

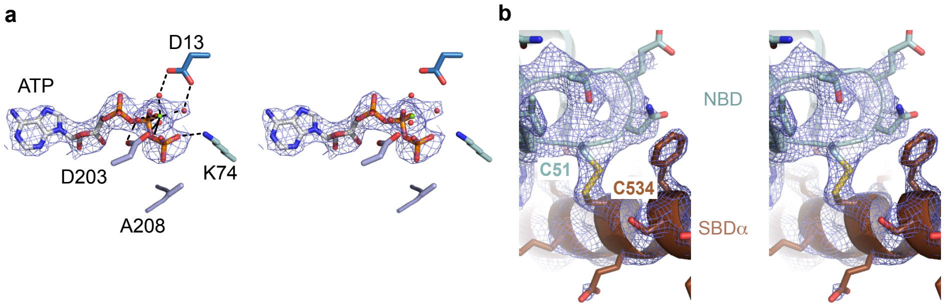

Supplementary Figure 4 | Electron density map of selected regions in the Ssb nucleotide binding domain. (**a**) Stereoview of ATP (sticks), Mg^{2+} (green ball) and catalytic residues (sticks) including the T208A mutation. The 2Fo-Fc electron density map is contoured at 1.75 σ. (**b**) 2Fo-Fc electron density map contoured at 1 σ for the disulfide bridge C51-C534 between NBD and SBDα.

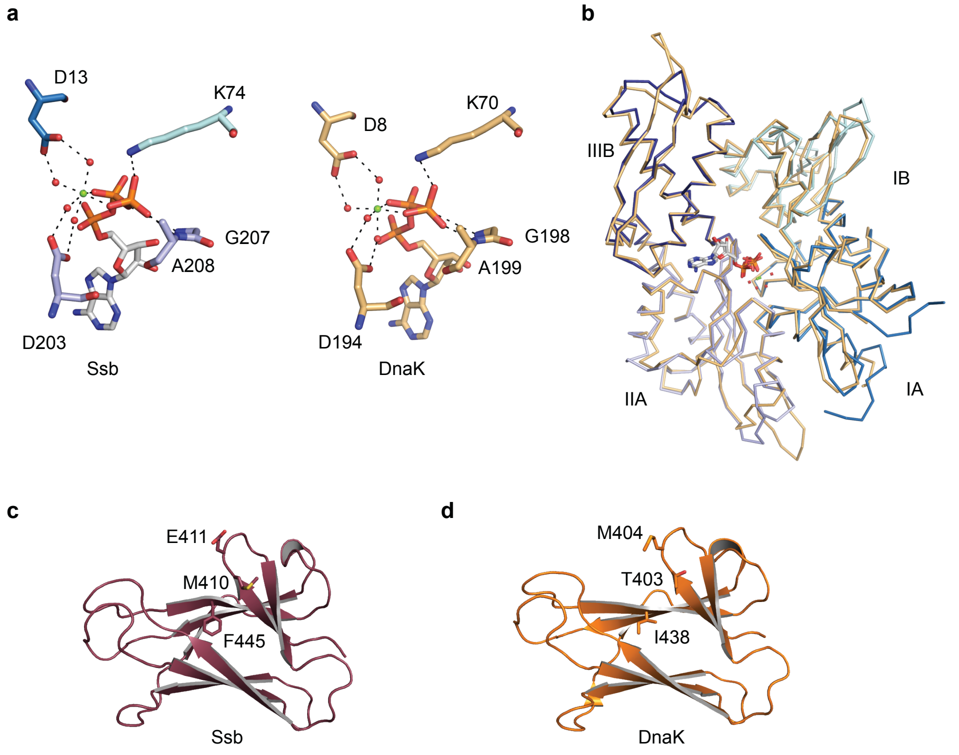

Supplementary Figure 5 | Structural comparison of Ssb and DnaK. (**a**) Comparison of the coordination of Mg^{2+} (green ball), ATP and catalytic residues in Ssb (left) and DnaK (right). (**b**) Superposition of the NBD of Ssb (blue color) with DnaK (PDB 4B9Q chain A, wheat color). (**c, d**) Comparison of the SBDβ of Ssb (purple color) with DnaK (wheat color).

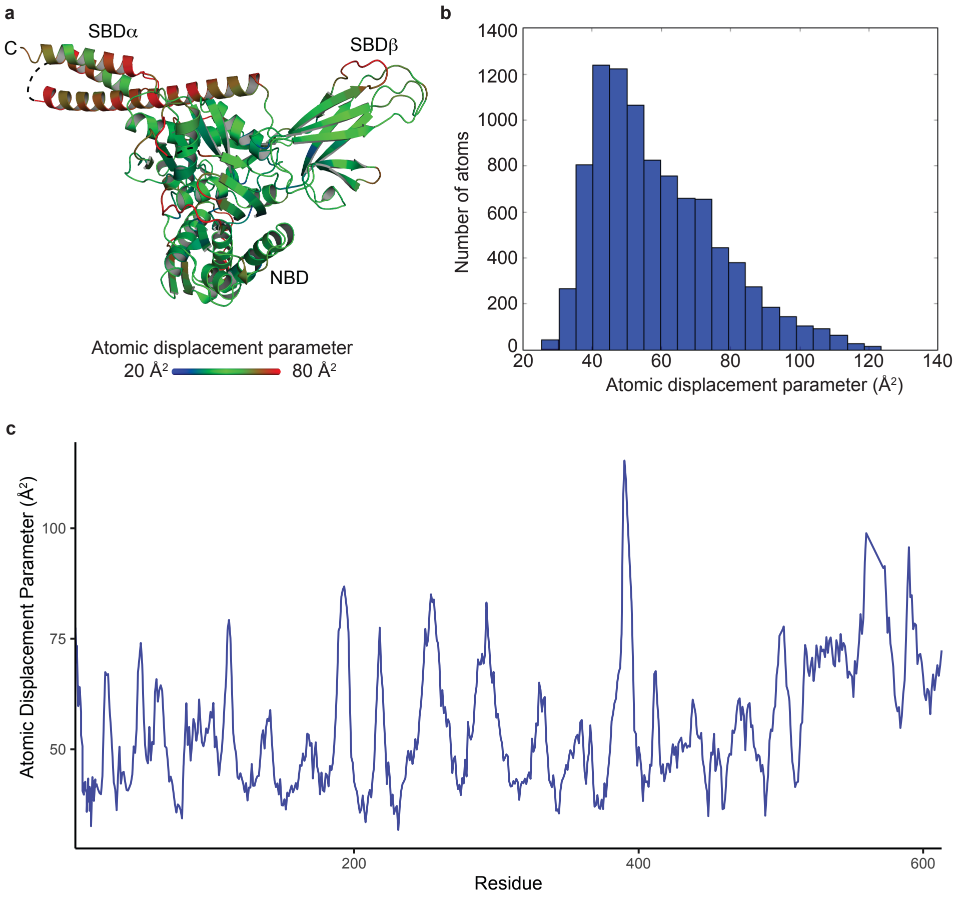

Supplementary Figure 6 | B-factor plot for the Ssb structure. (**a**) Structure of Ssb colored according to atomic displacement parameters (residue average), scaled from 20 (blue) to 80 (red) $Å^2$. (**b**) Distribution of atomic displacement parameters, generated with Phenix.validate[12] (**c**) Atomic displacement parameter derived dynamics. The atomic displacement parameters (residue average, calculated with BAVERAGE in CCP4[13]) are plotted as a function of the residue number for chain A.

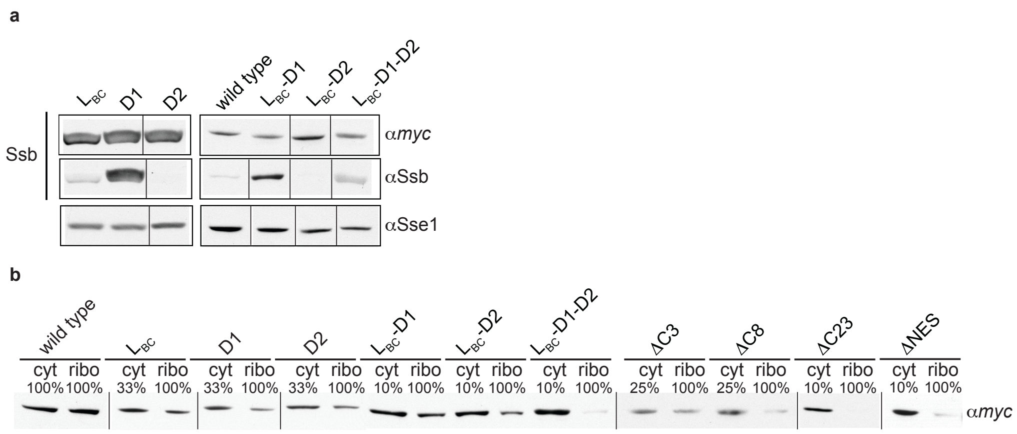

Supplementary Figure 7 │ Characterization of Ssb variants. (**a**) Mutations within the C-terminus of Ssb strongly affect recognition by αSsb. Total extract obtained from strains expressing *myc*Ssb1 variants as indicated were analyzed via immunoblotting employing either α*myc* or αSsb. Sse1 served as a control for equal loading. (**b**) Example blot employed for the quantification of the fraction of *myc*Ssb1 variants bound to the ribosome (Figure 3c). Total cell extract of yeast strains expressing wild type or mutant variants of Ssb were separated into a cytosolic fraction (cyt) and a ribosomal pellet (ribo) under low-salt conditions as described in methods. Details on the quantification are described in Supplementary methods.

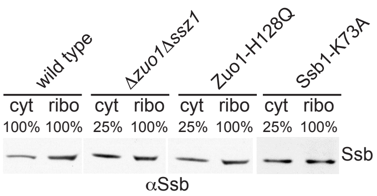

Supplementary Figure 8 │ Example blots for the quantification of ribosome-bound Ssb in Δ*zuo1*Δ*ssz1,* RAC-H128Q and Ssb1-K73A strains. Total cell extract of the strains indicated was separated into a cytosolic fraction (cyt) and a ribosomal pellet (ribo) under low-salt conditions as described in Methods. Details on the quantification are described in Supplementary methods.

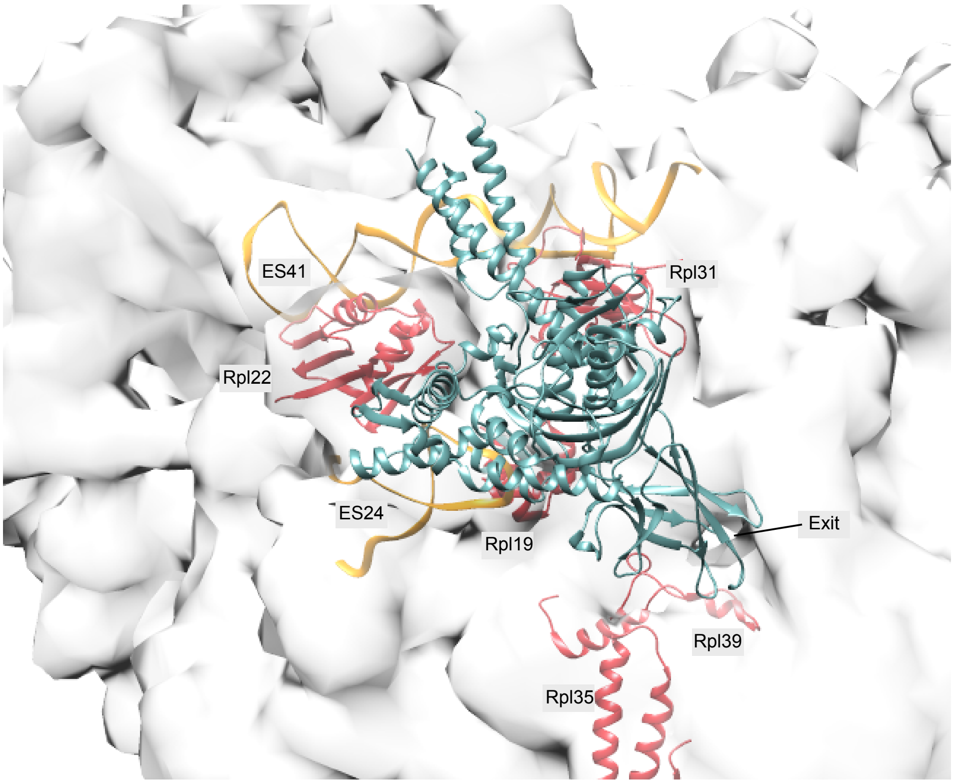

Supplementary Figure 9 │ Molecular model of Ssb at the ribosomal tunnel exit. Ssb in the pre-hydrolysis state (ATP-bound, open conformation) is placed on the ribosome. SBDβ locates close to the tunnel exit, while SBDα locates between Rpl22 and Rpl31, with the C-terminal positive patch close to ES41. Ribosomal proteins (red) and RNA (yellow) and Ssb (light blue) are shown as ribbon representation. Ribosomal surface is shown in white.

Figure 1a, original Western Blots

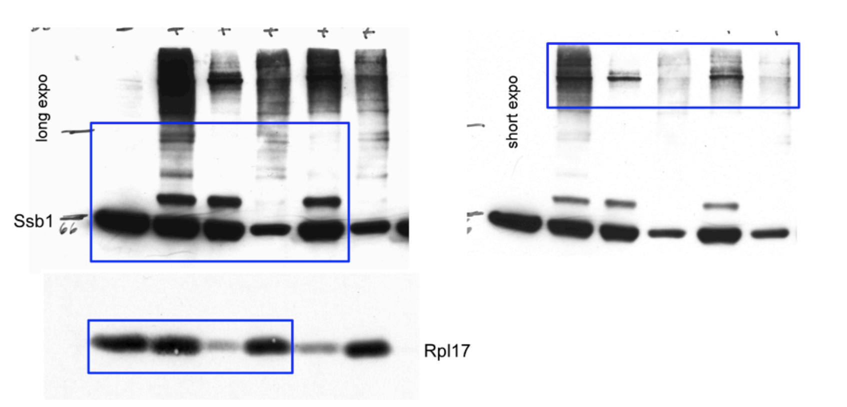

Figure 1c, original Western Blots

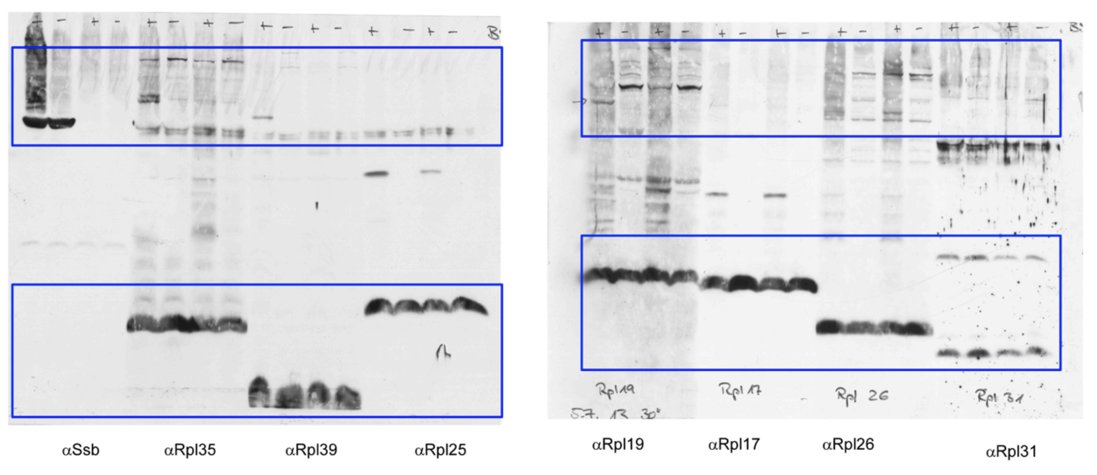

Supplementary Figure 10 | Uncropped images of Immunoblot. Blue boxes show cropped regions.

Figure 3c, original Western Blots

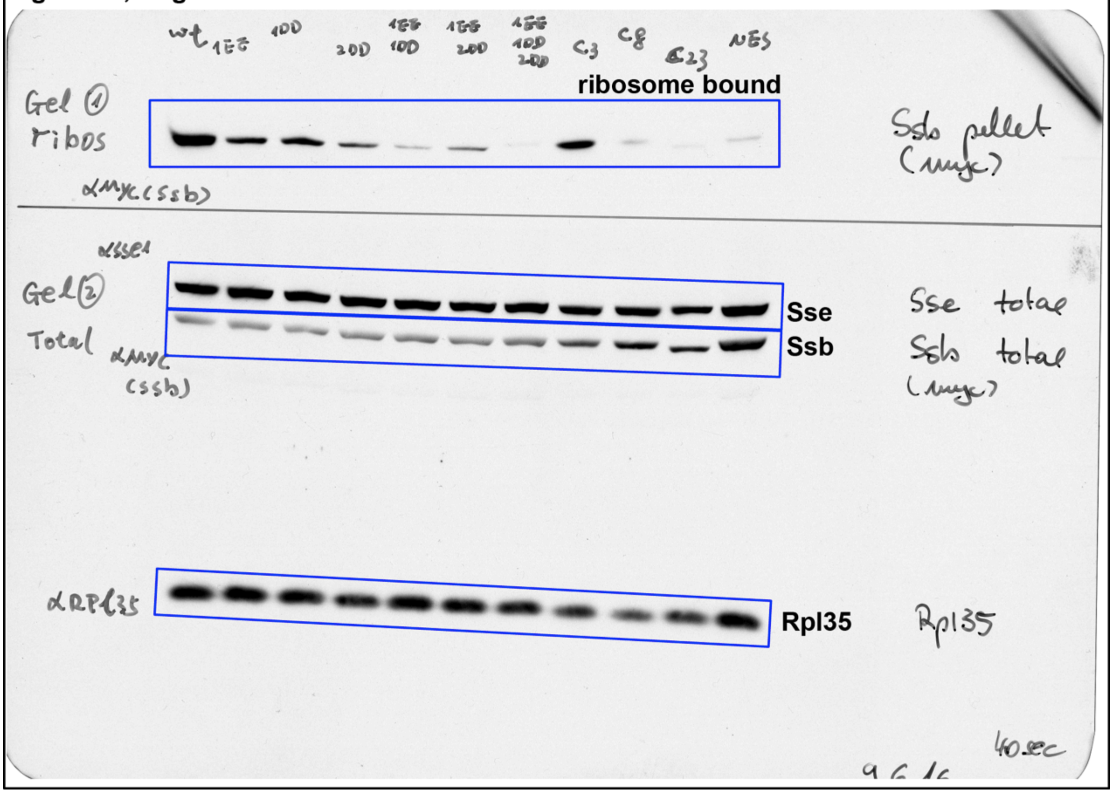

Supplementary Figure 11 | Uncropped images of Immunoblot. Blue boxes show cropped regions.

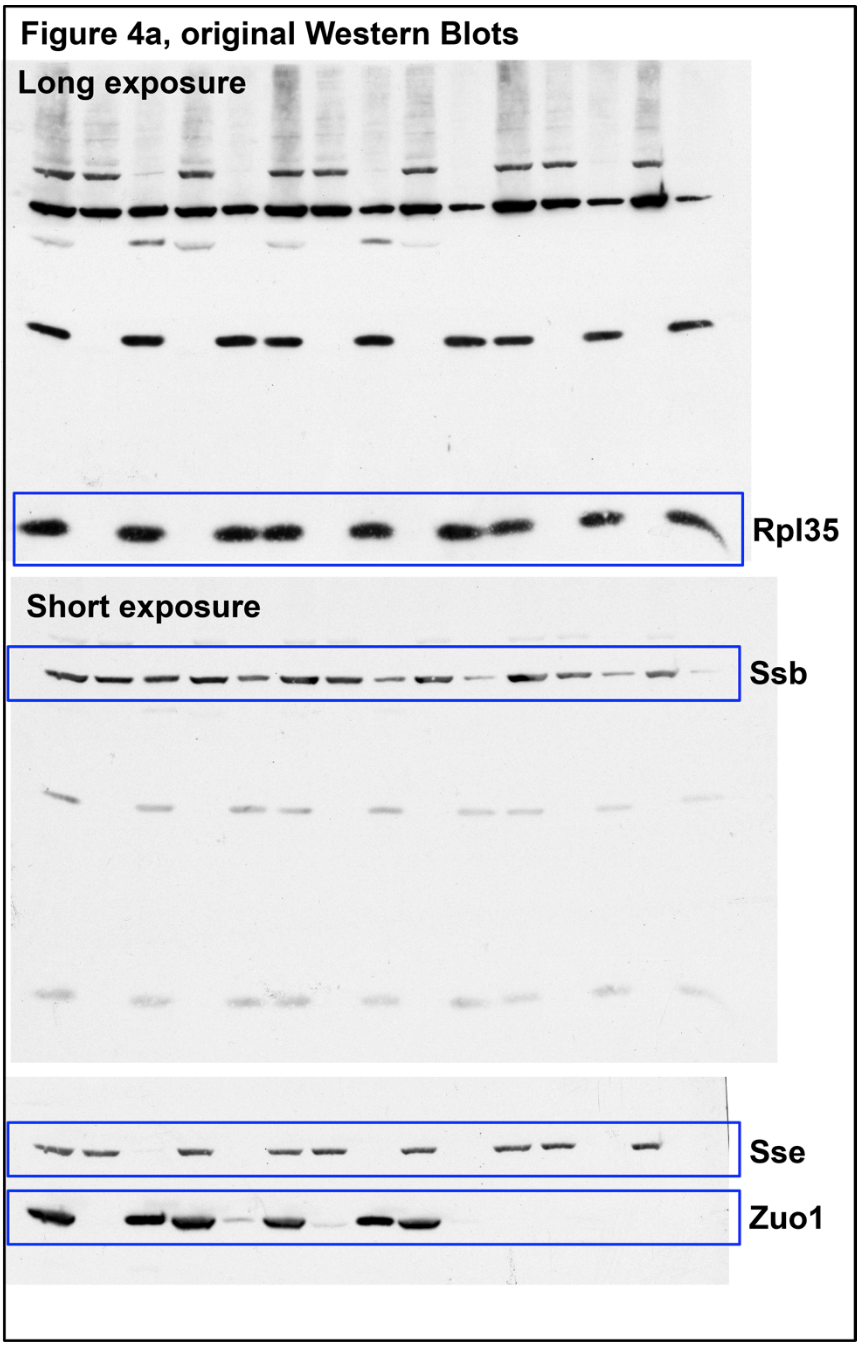

Supplementary Figure 12 | Uncropped images of Immunoblot. Blue boxes show cropped regions.

Figure 4a, original Western Blots

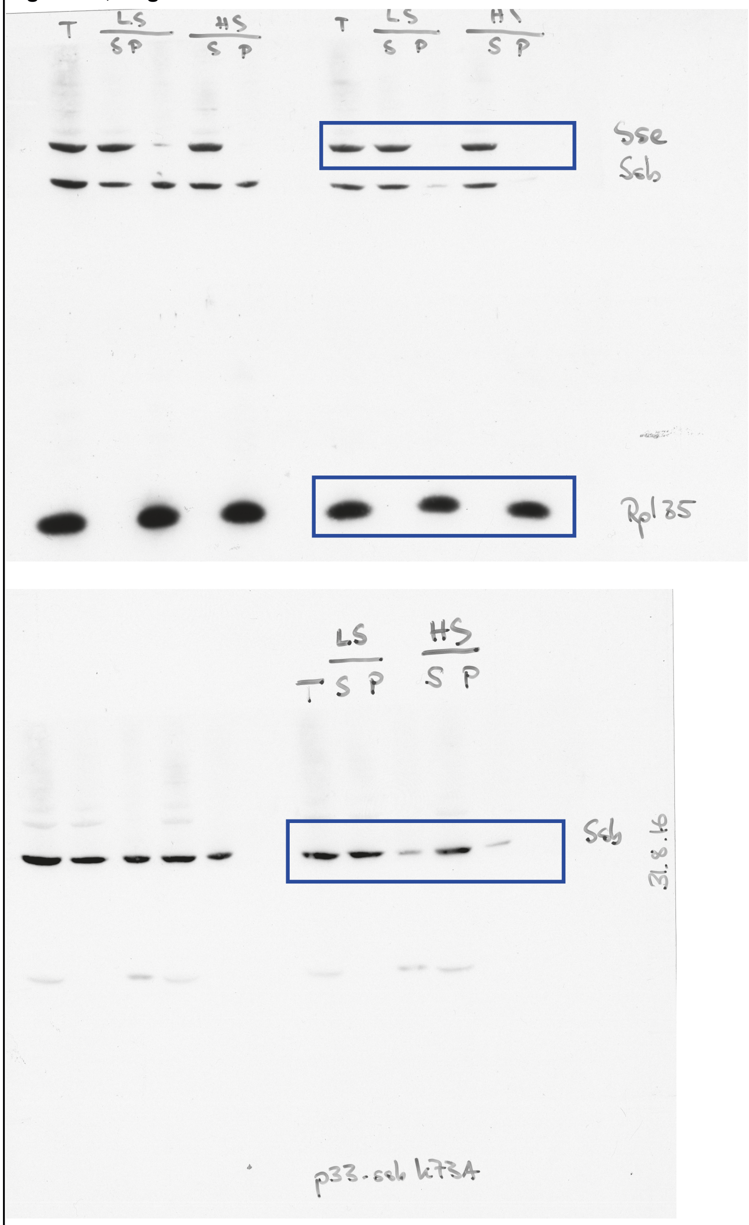

Supplementary Figure 13 | Uncropped images of Immunoblot. Blue boxes show cropped regions.

Figure 4a, original Western Blots

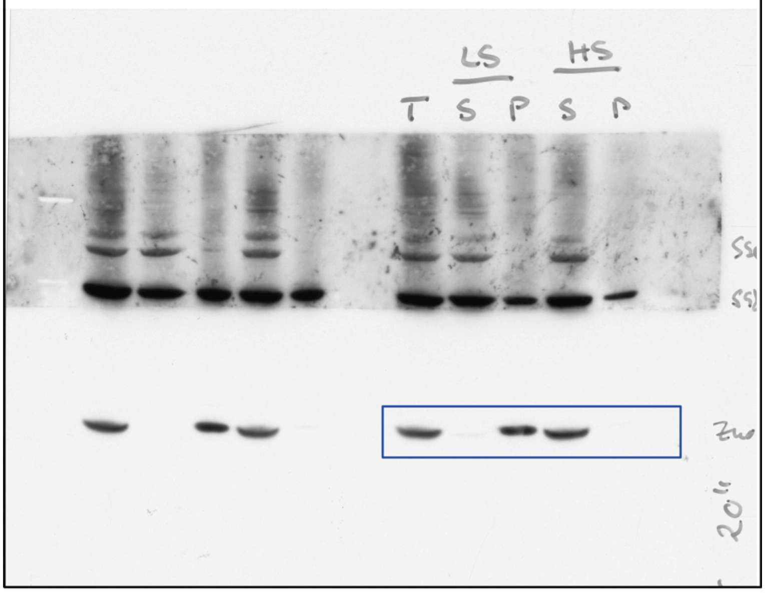

Figure 4c, original Western Blots

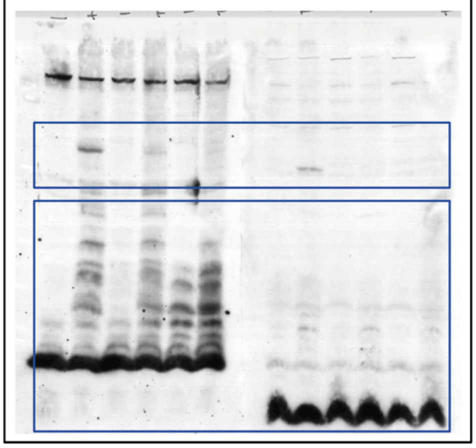

Supplementary Figure 14 | Uncropped images of Immunoblot. Blue boxes show cropped regions.

Figure 4d, original Western Blots

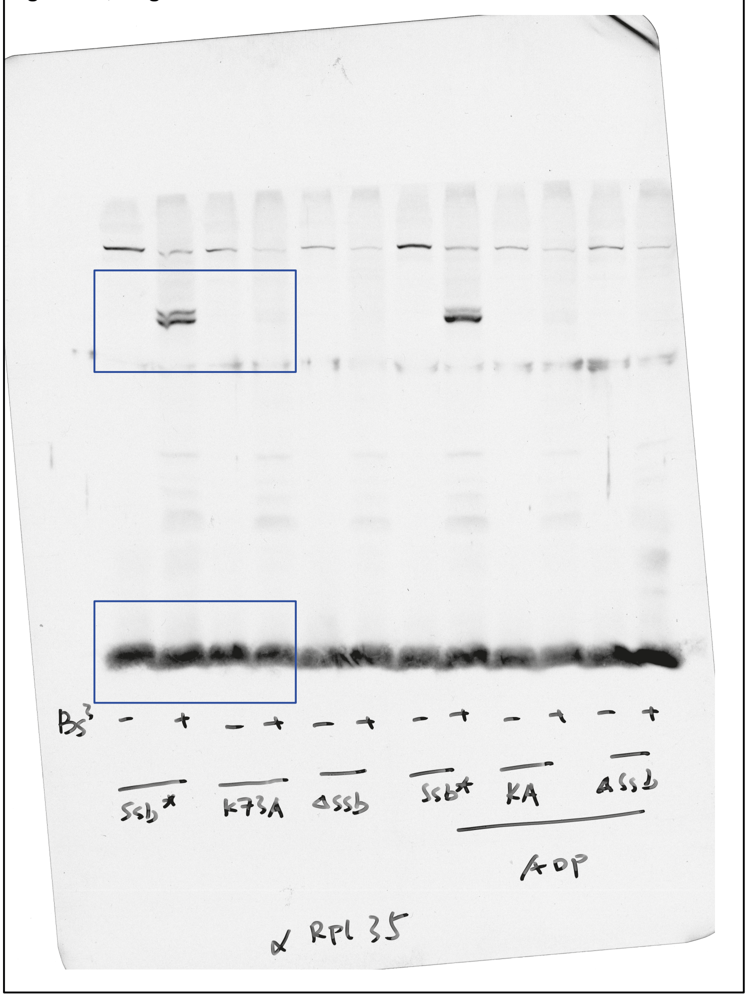

Supplementary Figure 15 | Uncropped images of Immunoblot. Blue boxes show cropped regions.

Figure 4d, original Western Blots

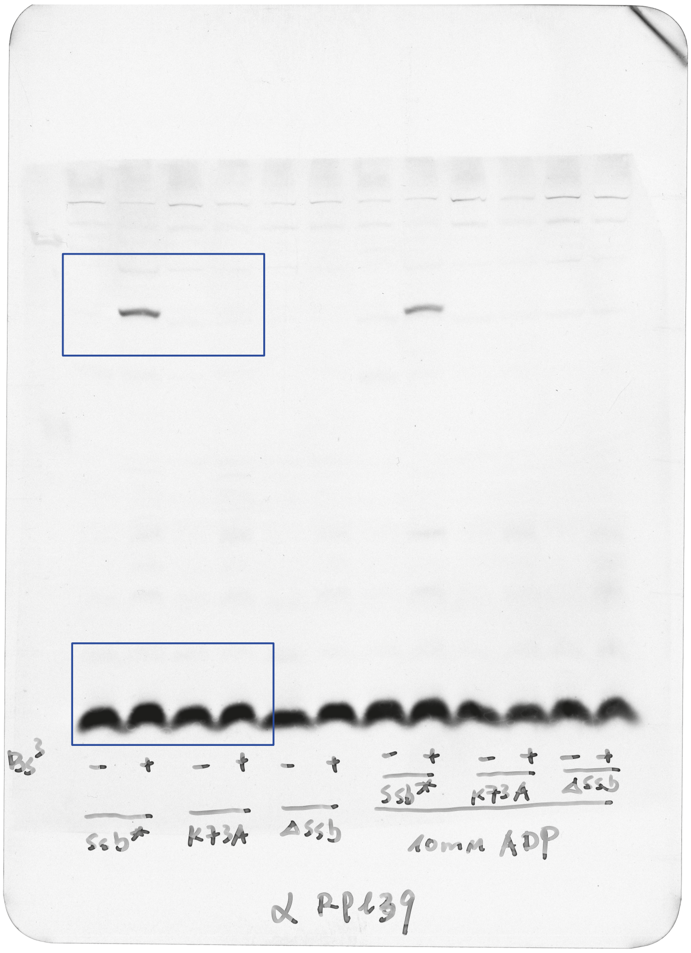

Supplementary Figure 16 | Uncropped images of Immunoblot. Blue boxes show cropped regions.

Supplementary Figure 1a, original Western Blots

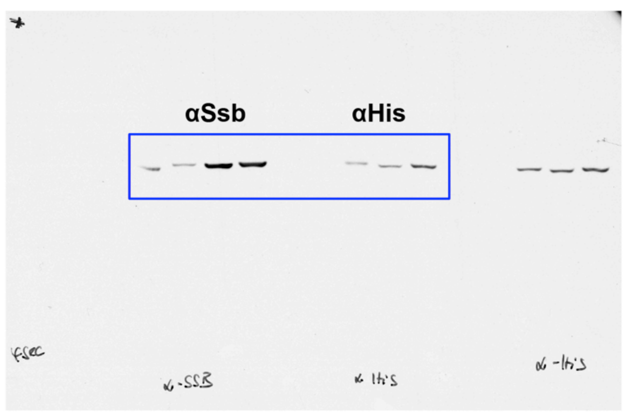

Supplementary Figure 1d, original Western Blots

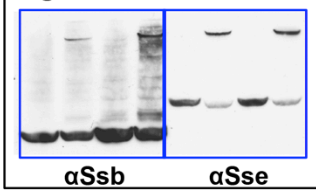

Supplementary Figure 1f, original Western Blots

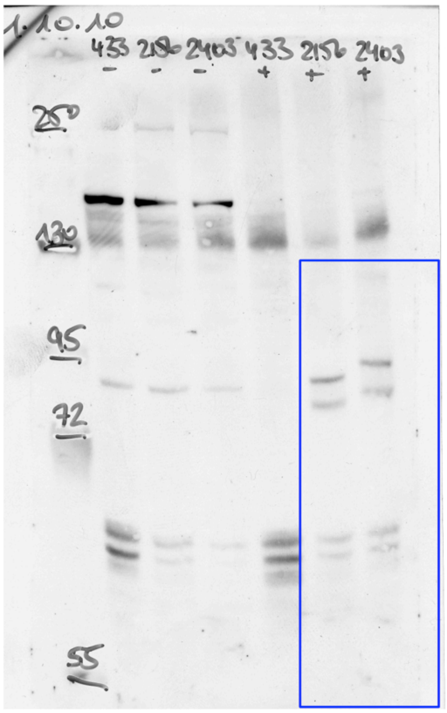

Supplementary Figure 17 | Uncropped images of Immunoblot. Blue boxes show cropped regions.

Supplementary Figure 1b, original Western Blots

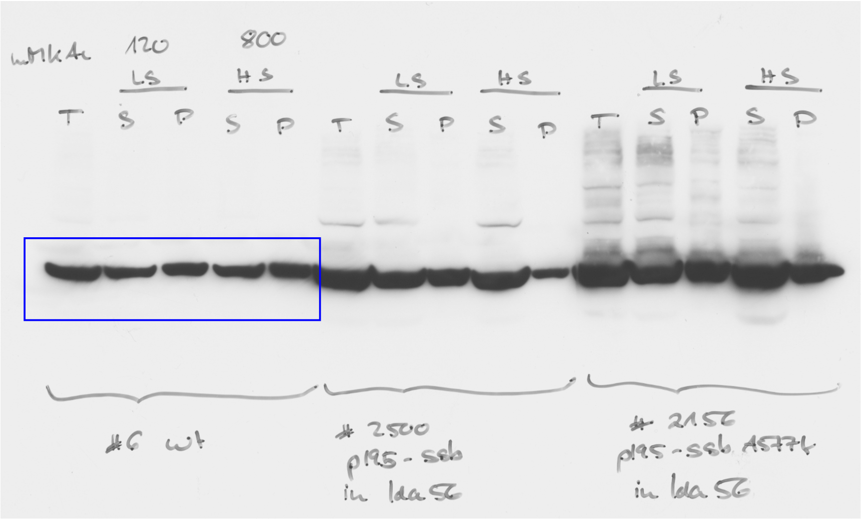

Supplementary Figure 1b, original Western Blots

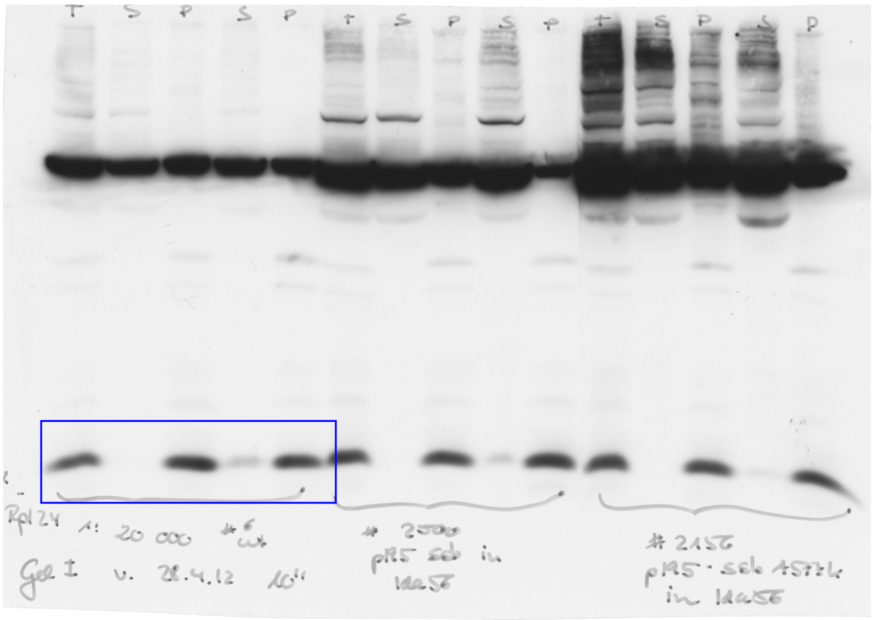

Supplementary Figure 18 | Uncropped images of Immunoblot. Blue boxes show cropped regions.

Supplementary Figure 1b, original Western Blots

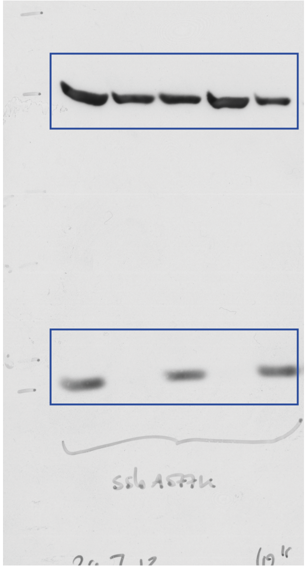

Supplementary Figure 1e, original Western Blots

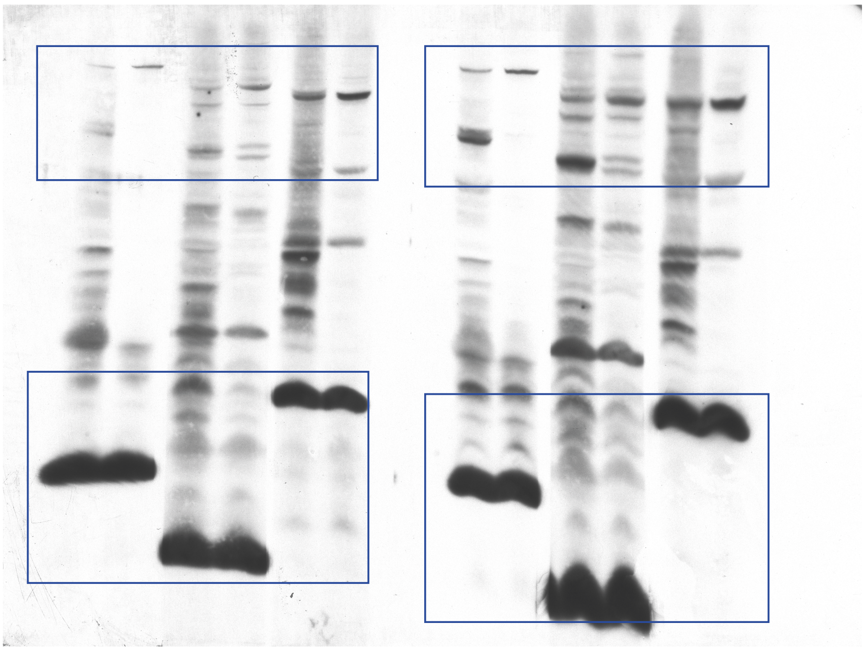

Supplementary Figure 19 | Uncropped images of Immunoblot. Blue boxes show cropped regions.

Supplementary Figure 7a, original Western Blots

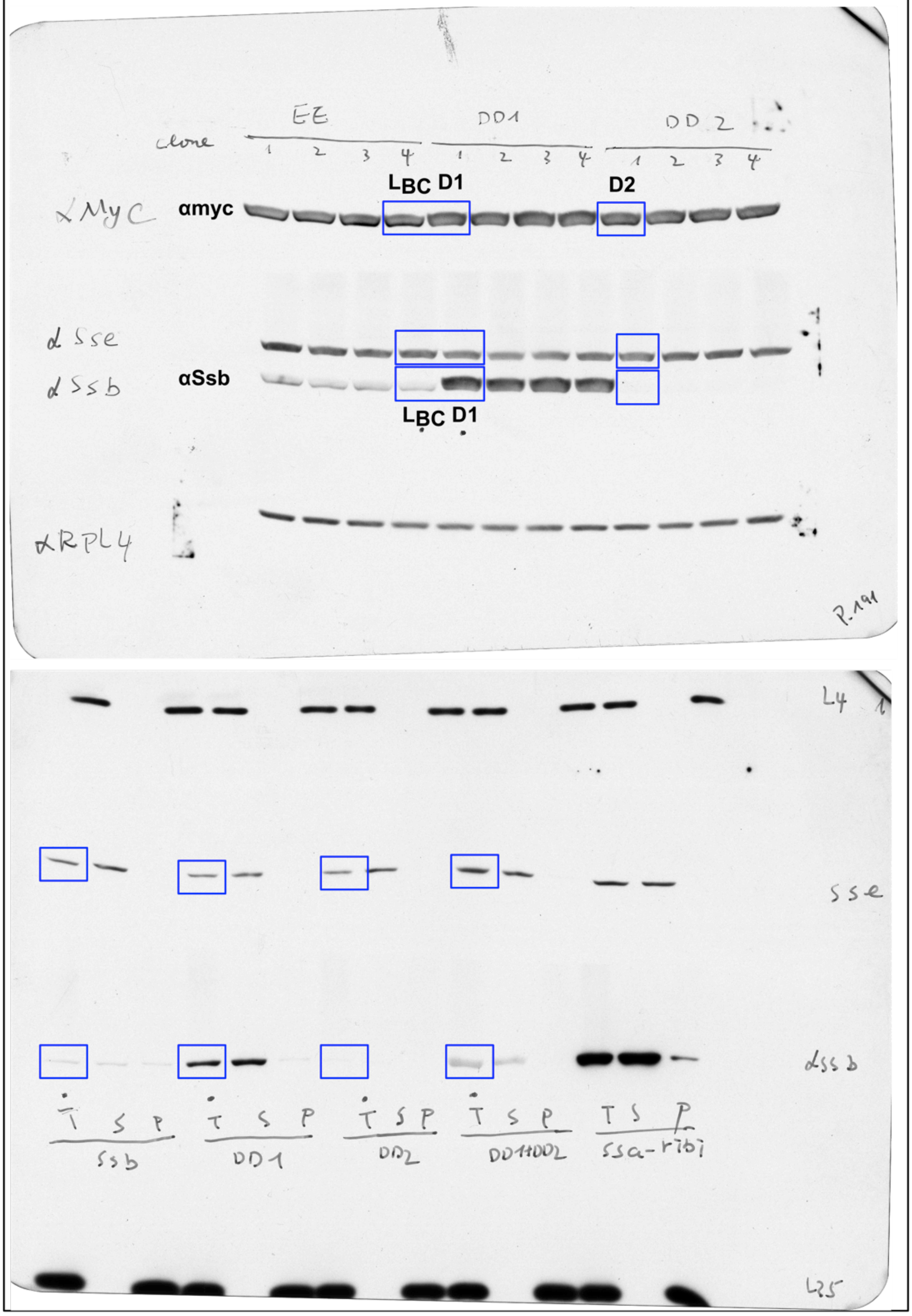

Supplementary Figure 20 | Uncropped images of Immunoblot. Blue boxes show cropped regions.

Supplementary Figure 7a, original Western Blots

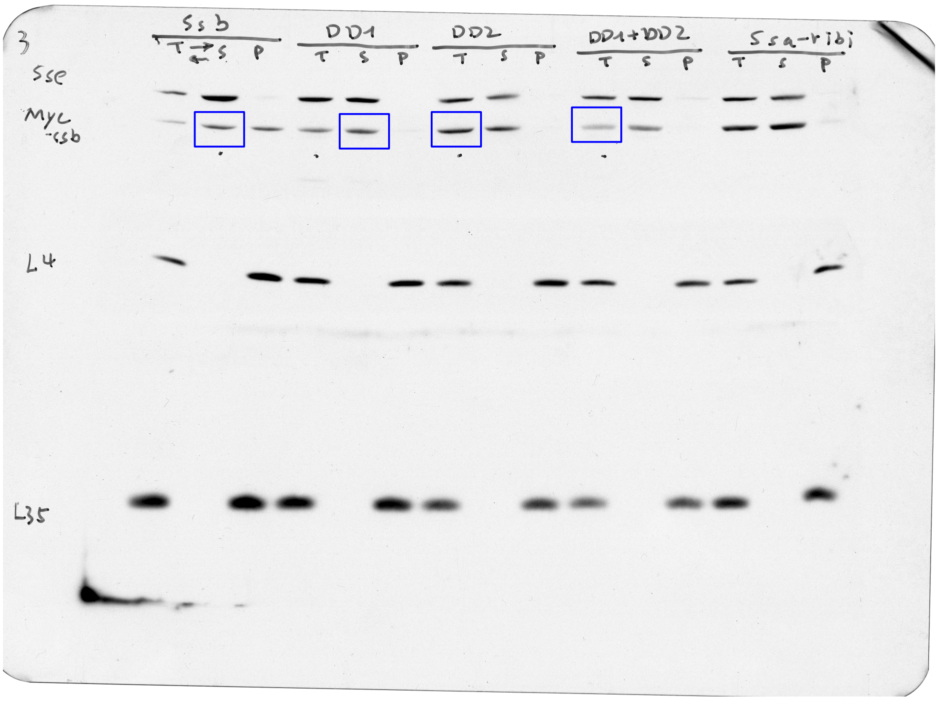

Supplementary Figure 21 | Uncropped images of Immunoblot. Blue boxes show cropped regions.

Supplementary Figure 7c, original Western Blots

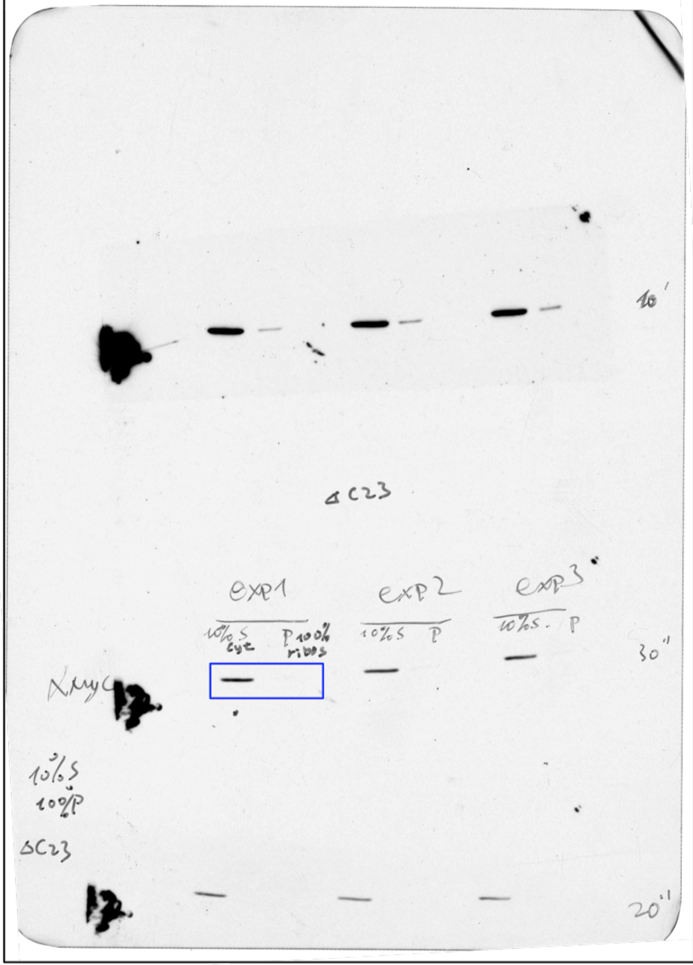

Supplementary Figure 22 | Uncropped images of Immunoblot. Blue boxes show cropped regions.

Supplementary Figure 7c, original Western Blots

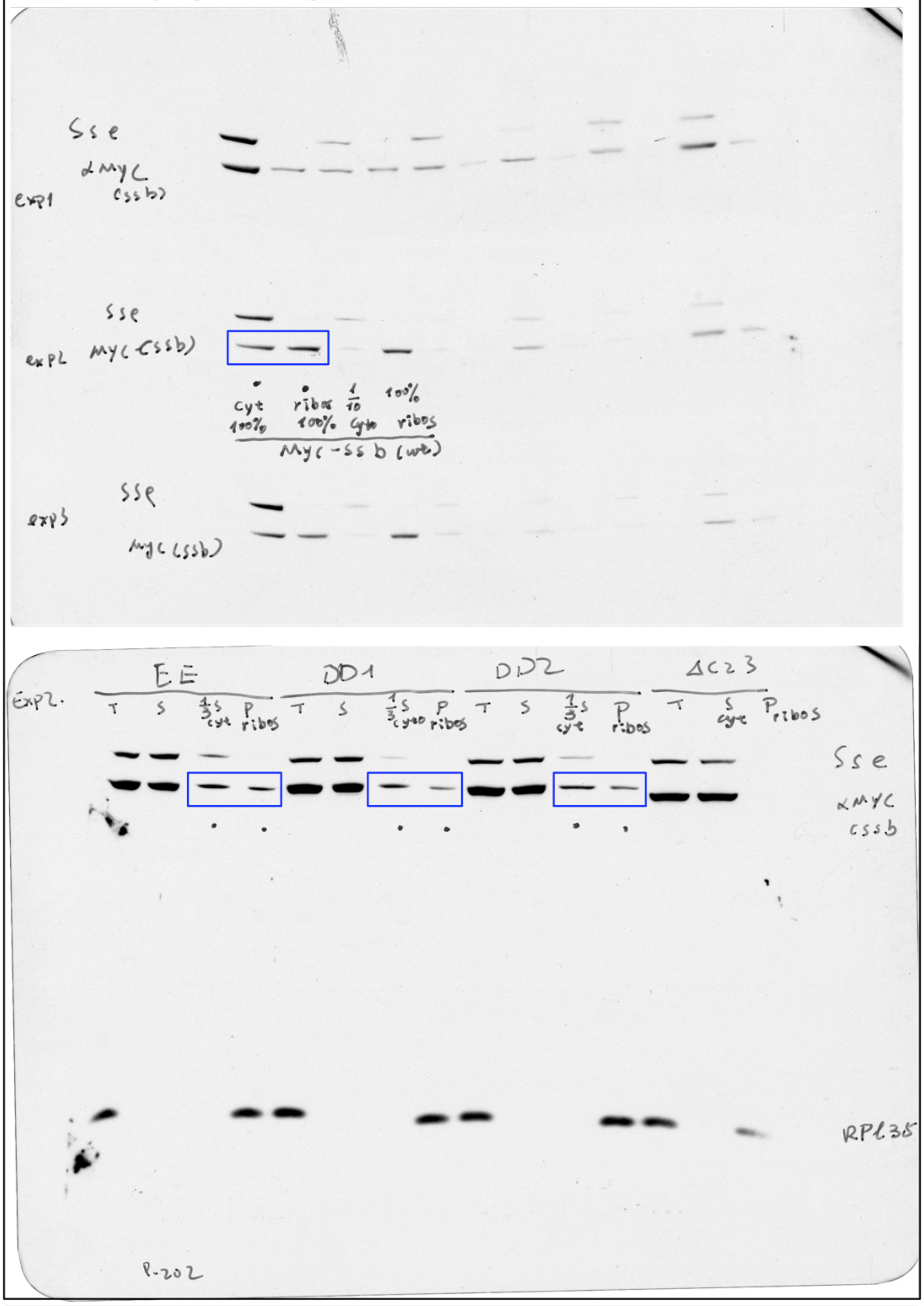

Supplementary Figure 23 | Uncropped images of Immunoblot. Blue boxes show cropped regions.

Supplementary Figure 7c, original Western Blots

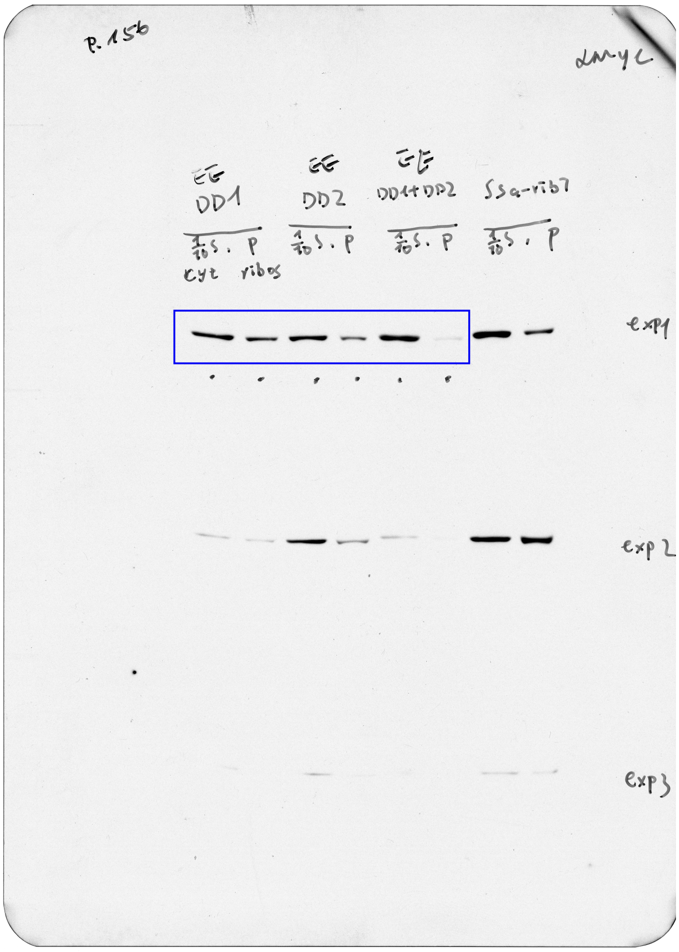

Supplementary Figure 24 | Uncropped images of Immunoblot. Blue boxes show cropped regions.

Supplementary Figure 7c, original Western Blots

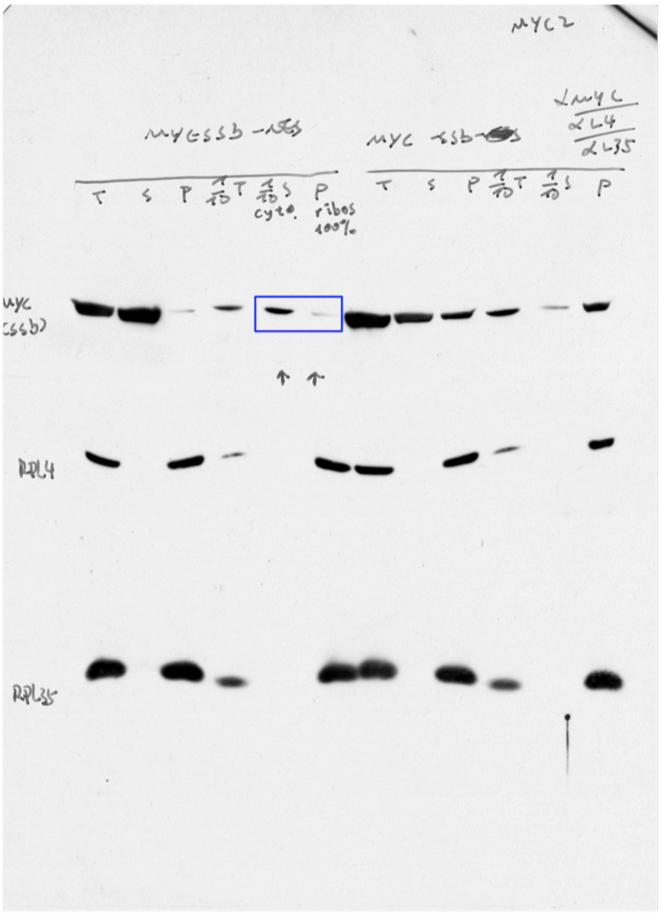

Supplementary Figure 25 | Uncropped images of Immunoblot. Blue boxes show cropped regions.

Supplementary Figure 7c, original Western Blots

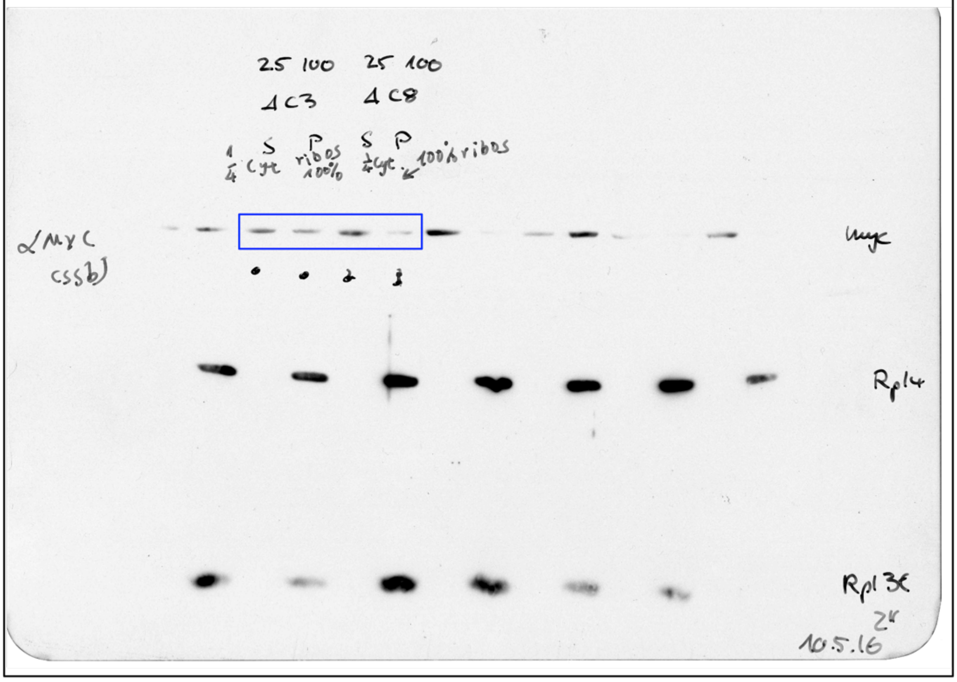

Supplementary Figure 26 | Uncropped images of Immunoblot. Blue boxes show cropped regions.

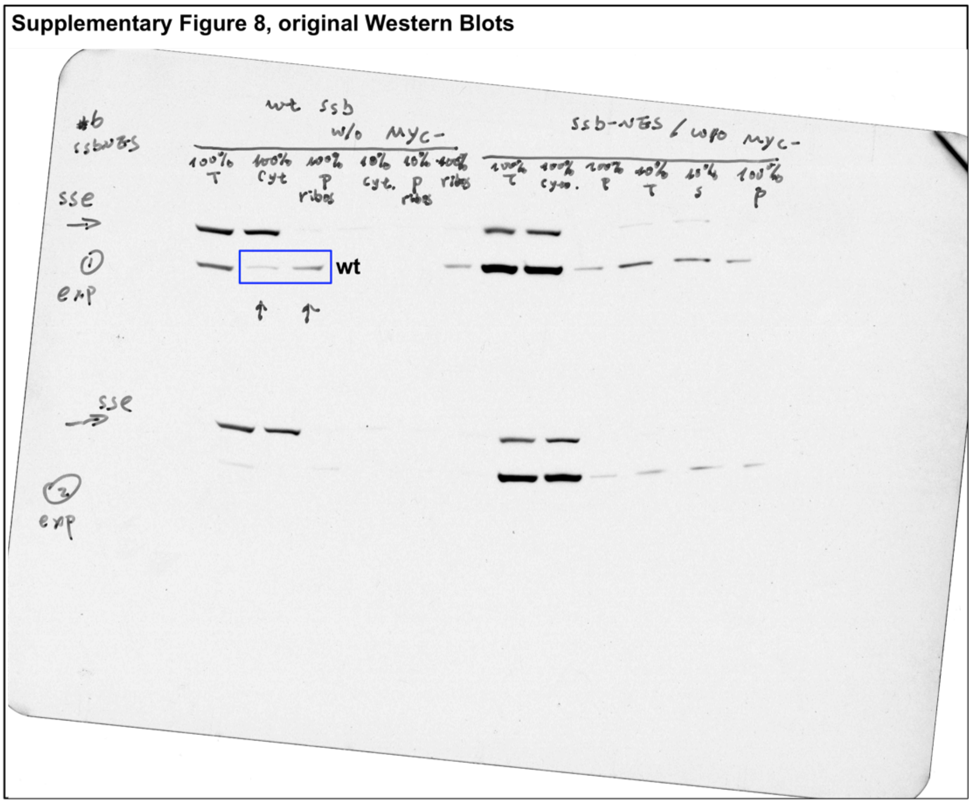

Supplementary Figure 27 | Uncropped images of Immunoblot. Blue boxes show cropped regions.

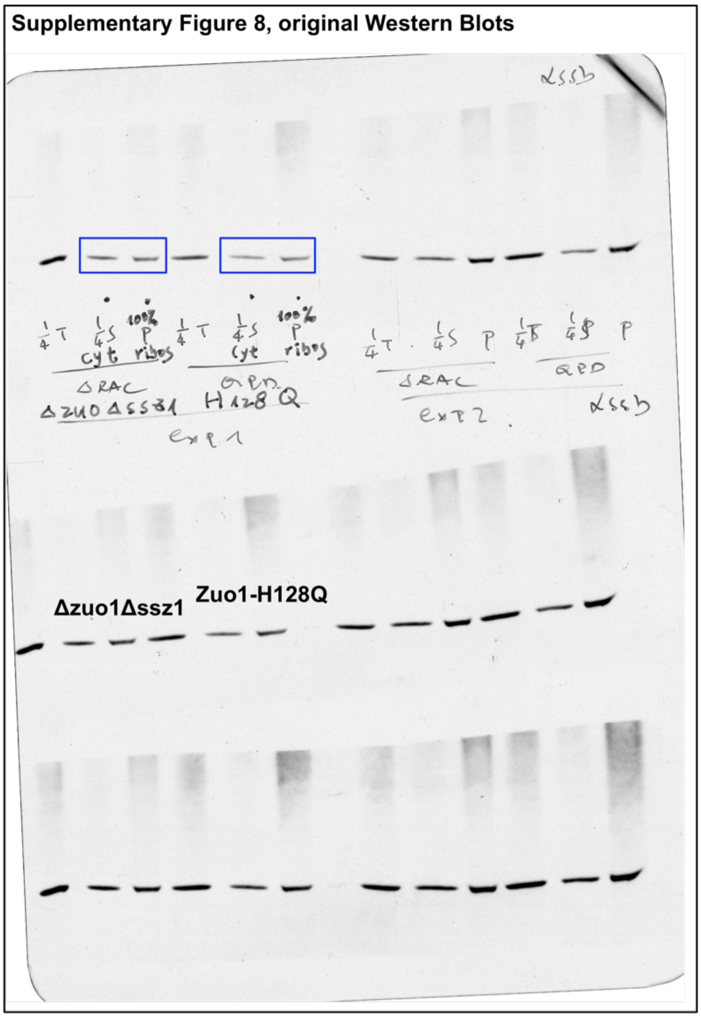

Supplementary Figure 28 | Uncropped images of Immunoblot. Blue boxes show cropped regions.

Supplementary Figure 8, original Western Blots

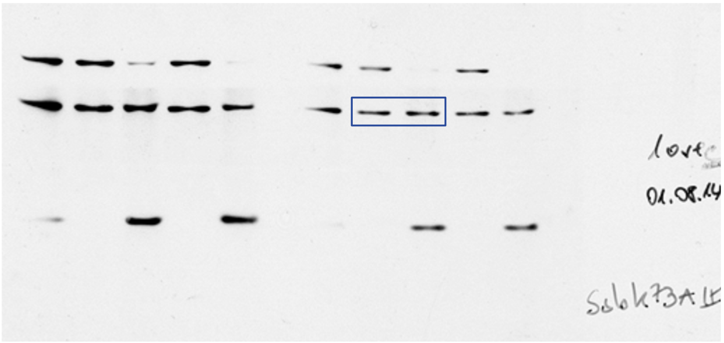

Supplementary Figure 29 | Uncropped images of Immunoblot. Blue boxes show cropped regions.

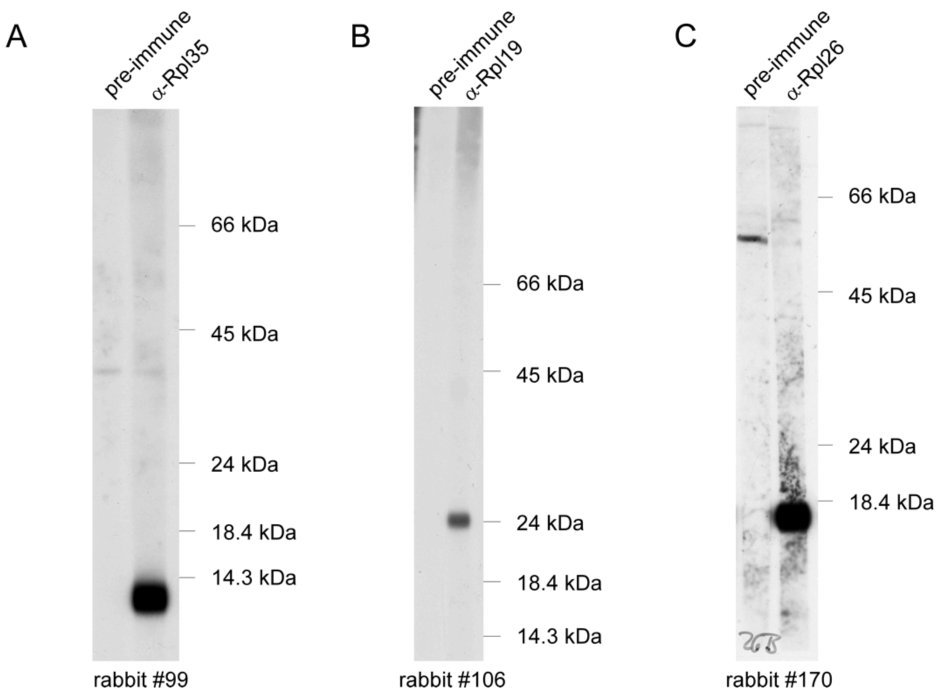

Supplementary Figure 30 | Validation of αRpl35, αRpl19 and αRpl26 antibodies Peptide antibodies directed against the ribosomal proteins **(a)** Rpl35 (13.9 kDa), **(b)** Rpl19 (21.7 kDa), and **(c)** Rpl26 (14.3 kDa) were raised in rabbits (EUROGENTEC, Bel S.A). Total yeast protein extract was separated via SDS-PAGE on Tris-Tricine gels, which were blotted onto nitrocellulose membranes. Subsequently the membranes were cut into strips, which were decorated with either pre-immune, or the final bleed of the same rabbit. Immunoblots were developed using ECL as described in the Methods section. Uncropped blots are shown in supplementary figures 31-32.

Supplementary Figure 30, original Western Blots

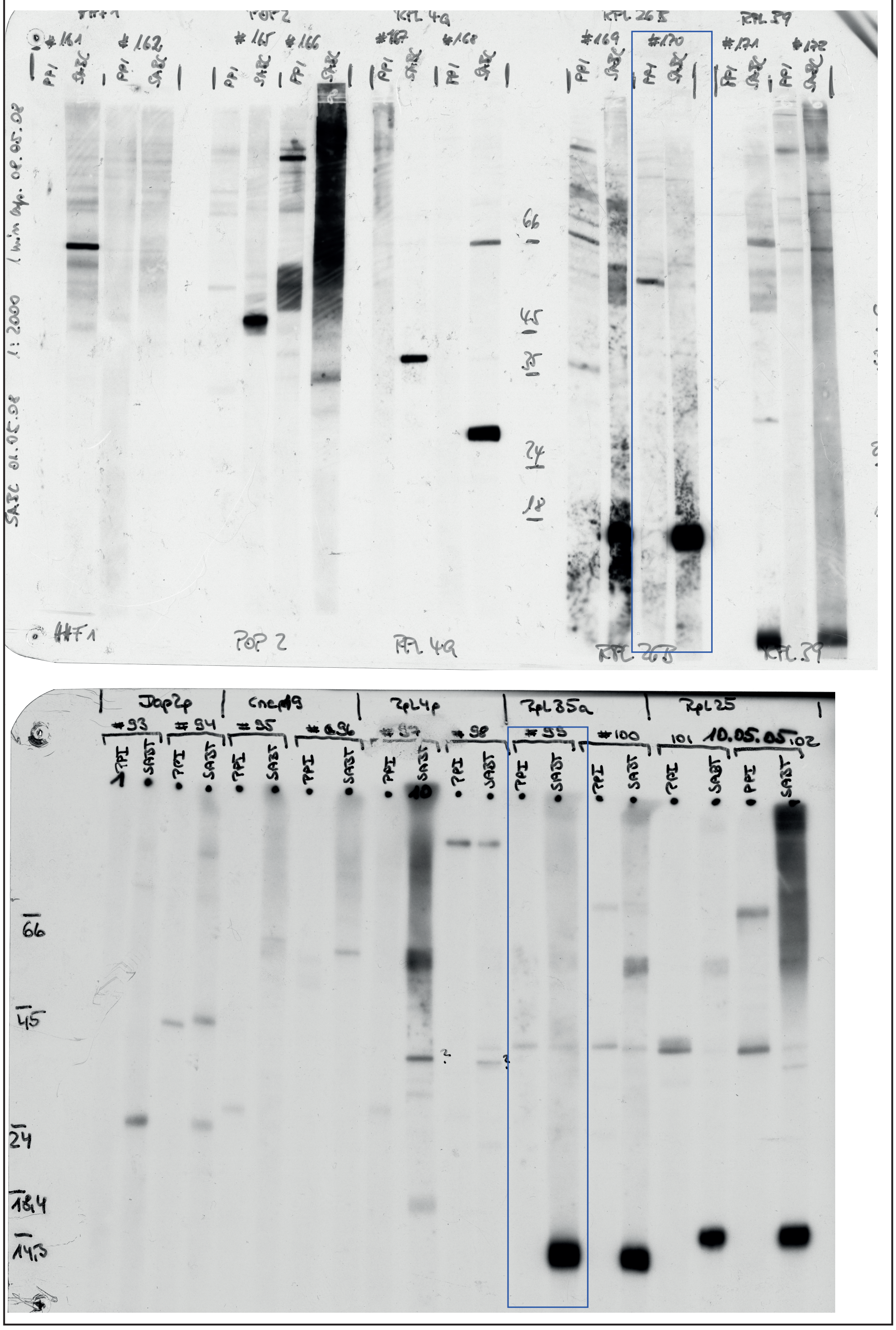

Supplementary Figure 31 | Uncropped images of Immunoblot. Blue boxes show cropped regions.

Supplementary Figure 30, original Western Blots

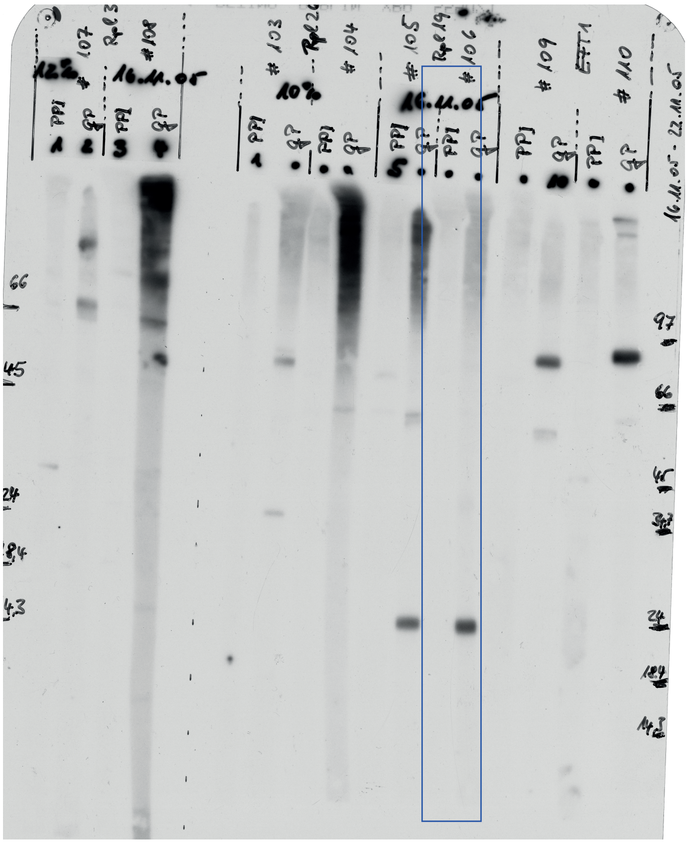

Supplementary Figure 32 | Uncropped images of Immunoblot. Blue boxes show cropped regions.

Supplementary Table 1 | Strains used in this study.

Name	Genotype	Reference
S. cerevisiae		
BY4742	MATα *his3 leu2 lys2 ura3*	[1]
MH272-3f	MATa/α *ura3/ura3 leu2/leu2 his3/his3 trp1/trp1 ade2/ade2*	[2]
BY4742::ProtA-TEV-His_6-Ssb1	ProtA-TEV-His_6:Ssb1:clonNAT	This work
Δ*ssb1*Δ*ssb2*	*ssb1::ADE2 ssb2::HIS3*	[3]
Δ*ssb1*Δ*ssb2*	*ssb1::kanMX ssb2::HIS3*	[4]
Δ*ssb1*Δ*ssb2*	*ssb1::ADE2 ssb2::ADE2*	[5]
Δ*zuo1*Δ*ssz1*	*zuo1::TRP1 ssz1::LEU2*	[6]
Δ*ssb1*Δ*ssb2*Δ*zuo1*	*ssb1::ADE2 ssb2::ADE2 zuo1::TRP1*	This work
Δ*ssb1*Δ*ssb2*Δ*zuo1*Δ*ssz1*	*ssb1::ADE2 ssb2::ADE2 zuo1::TRP1 ssz1::LEU2*	[7]
RAC-H128Q	*zuo1::TRP1* + pYEPlac33-Zuo1-H128Q	[6]
RAC-H128Q Ssb1*	*ssb1::ADE2 ssb2::ADE2 zuo1::TRP1* + pYEPlac181-zuoQPD + pYEPlac195-Ssb1-A577K	This work
Ssb1*	*ssb1::ADE2 ssb2::HIS3* + pYEPlac195-Ssb1-A577K	This work
Ssb1-K73A	*ssb1::ADE2 ssb2::HIS3* + pYCPlac33-Ssb-K73A	[5]
Ssb1*-K73A	*ssb1::ADE2 ssb2::HIS3* + pYEPlac195-Ssb1-A577K	this work
Δ*zuo1*Δ*ssz1* Ssb1*	*ssb1::ADE2 ssb2::ADE2 zuo1::TRP1 ssz1::LEU2* + pYEPlac195-Ssb1-A577K	This work
*myc*Ssb1 (and *myc*Ssb1 variants)	*ssb1::ADE2 ssb2::HIS3* + pCM190-mycSsb1	This work
Δ*rpl35a*Δ*rpl35b* a/α	*RPL35a/rpl35a::TRP1 RPL35b/rpl35b::LEU2*	This work
Δ*rpl35a*Δ*rpl35b* + Rpl35a	*rpl35a::TRP1 rpl35b::LEU2* + *pYCPlac33-Rpl35a*	This work
Δ*rpl35a*Δ*rpl35b* + Rpl35a-FLAG Ssb1*	*rpl35a::TRP1 rpl35b::LEU2 ssb1::kanMX ssb2::HIS3* + *pYCPlac444-Rpl35a-FLAG* + *pYEPlac195-Ssb1A577K*	This work
Ssb1	*ssb1::ADE2 ssb2::HIS3* + pYCPlac33-Ssb1	This work
Ssb1*	*ssb1::ADE2 ssb2::HIS3* + pYCPlac33-Ssb1-A577K	This work
His-Ssb1	*ssb1::ADE2 ssb2::HIS3* + pESC-Ura-His_6Ssb1	This work
His-Ssb1-ΔNES	*ssb1::ADE2 ssb2::HIS3* + pESC-Ura-His_6Ssb1-ΔNES	This work
His-Ssb1*	*ssb1::ADE2 ssb2::HIS3* + pESC-Ura-His_6Ssb1-ΔNES	This work
E. coli		
BL21(DE3) Rosetta2	F^- *ompT hsdS$_B$*(r_B^- m_B^-) *gal dcm* λ(DE3) *tonA* pRARE2 (Cam^R)	Novagen

Supplementary Table 2 | Plasmids used in this study.

Name	Description	Reference
pET24a	Expression vector, Kan^R	Novagen
pET24a-His_6-ctSsb-E51C-T208A-D534C	Kan^R, E51C, T208A and D534C	This work
pET24a-His_6-ctSsb536-614	Kan^R, Ssb residues A536 to R614	This work
pET24a-His_6-ctSsb536-614ΔNES	Kan^R, deletion IEQALSEAM	This work
pET24a-His_6-ctSsb536-614-L_{BC}	Kan^R, K568E and R569E	This work
pET24a-His_6-ctSsb536-614-D1	Kan^R, K597D and K598D	This work
pET24a-His_6-ctSsb536-614-D2	Kan^R, K604D and R605D	This work
pET24a-His_6-ctSsb536-614-L_{BC}-D1	Kan^R, K568E, R569E, K597D and K598D	This work
pET24a-His_6-ctSsb536-614-L_{BC}-D2	Kan^R, K568E, R569E, K604D and R605D	This work
pET24a-His_6-ctSsb536-614-L_{BC}-D1-D2	Kan^R, K568E, R569E ,K597D,K598D, K604D and R605D	This work
pYCPlac33-Ssb1	*ARS1-CEN4*, *URA3*	This work
pYCPlac33-Ssb1-A577K (Ssb1*)	*ARS1-CEN4*, *URA3*	This work
pYCPlac33-Zuo1-H128Q	*ARS1-CEN4*, *URA3*	[6]
pYCPlac33-Ssb1-K73A	*ARS1-CEN4*, *URA3*	[5]
pYEPlac195-Ssb1-K73A-A577K	2µ URA3	This work
pYEPlac195-Ssb1-A577K	2 µ, *URA3*	This work
pYEPlac181-Zuo1-H128Q	2 µ, *LEU2*	This work
pYCPlac33-Rpl35a	*ARS1-CEN4*	This work
pYCPlac444	*ARS1-CEN4*	This work
pYCPlac444-Rpl35a-FLAG	*ARS1-CEN4*, *ADE2*	This work
pESC-Ura-His_6Ssb1	2 µ, *URA3,* expression under control of the GAL10 promoter	This work
pESC-Ura-His_6Ssb1-ΔNES	2 µ, *URA3,* expression under control of the GAL10 promoter	This work
pESC-Ura-His_6Ssb1-A577K (His_6Ssb1*)	2 µ, *URA3,* expression under control of the GAL10 promoter	This work
pCM190-*myc*Ssb1	2 µ, *URA3*	This work
pCM190-*myc*Ssb1-L_{BC}	2 µ, *URA3,* K567E and R568E	This work
pCM190-*myc*Ssb1-D1	2 µ, *URA3,* R596D and K597D	This work
pCM190-*myc*Ssb1-D2	2 µ, *URA3,* K603D and R604D	This work
pCM190-*myc*Ssb1-L_{BC}-D1	2 µ, *URA3,* K567E,R568E, R596D, and K597D	This work
pCM190-*myc*Ssb1-L_{BC}-D2	2 µ, *URA3,* K567E, R568E, K603D, R604D	This work
pCM190-*myc*Ssb1-L_{BC}-D1-D2	2 µ, *URA3,* K567E, R568E, R596D, K597D, K603D, R604D	This work
pCM190-*myc*Ssb1-ΔC3	2 µ, *URA3,* deletion of residues 611-613	This work
pCM190-*myc*Ssb1-ΔC8	2 µ, *URA3,* deletion of residues 606-613	This work
pCM190-*myc*Ssb1-ΔC23	2 µ, *URA3,* deletion of residues 591-613	This work
pCM190-*myc*Ssb1-ΔNES	2 µ, *URA3,* deletion of residues 574-586	This work

Supplementary Table 3 | Nomenclature of ribosomal proteins used in this study.

Yeast name used	New name [8]
Rpl17	uL22
Rpl19	eL19
Rpl22	eL22
Rpl24	eL24
Rpl25	uL23
Rpl26	uL24
Rpl31	eL31
Rpl35	uL29
Rpl39	eL39

Supplementary methods

Strains and plasmids. N-terminally His-tagged (GSSHHHHHHSS) versions of Ssb1, Ssb1-ΔNES, and Ssb1* were generated by PCR technology and were cloned into pESCUra (Agilent Technologies) under control of the *GAL10* promoter. *RPL35a* +/- 500 bp up- and down-stream was cloned into the EcoRI/SalI of pYCPlac33[9] resulting in pYCPlac33-Rpl35a. pYCPlac33-FLAG-Rpl35a was constructed by fusing the FLAG-tag (DYKDDDDK) to the C-terminus of Rpl35a via PCR technology. Gene disruption cassettes were generated by replacing an internal 273 bp HindIII fragment in exon 2 of *RPL35a* with the *TRP1* marker, or, in case of *RPL35b with the LEU2* marker. The disruption constructs were used to generate the diploid *RPL35a*/*rpl35a*::*TRP1 RPL35b*/*rpl35b*::*LEU2* strain. Because the Δ*rpl35a*Δ*rpl35b* double deletion is lethal[10] the diploid strain was transformed with pYCPlac33-Rpl35a prior to sporulation and tetrad analysis. A haploid *rpl35a*::*TRP1 rpl35b*::*LEU2* strain complemented by pYCPlac33-Rpl35a (Δ*rpl35a*Δ*rpl35b* + Rpl35a) was selected for further experiments. The quadruple deletion strain Δ*ssb1*Δ*ssb2*Δ*rpl35a*Δ*rpl35b* + Rpl35a was generated by mating Δ*ssb1*Δ*ssb2* (*ssb1::kanR ssb2::HIS3*)[6] with Δ*rpl35a*Δ*rpl35b* + Rpl35a followed by sporulation and tetrad analysis. FLAG-RPL35 was cloned into pYCPlac444, a derivate of pYCPlac111[9] in which the *LEU2* marker gene was replaced with *ADE2* marker gene and pYCPlac33-Rpl35a was replaced by FLAG-Rpl35a via plasmid shuffling[11].

Quantification of the fraction of ribosome-bound Ssb via immunoblotting. Total cell extract of yeast strains expressing wild type or mutant variants of Ssb were separated into a cytosolic fraction (cyt) and a ribosomal pellet (ribo) under low-salt conditions as described in Methods. To obtain band intensities on immunoblots in the linear range, loading of the cytosolic and ribosomal fractions was adjusted such that the Ssb-band in the cytosolic and in the ribosomal fractions was in the same intensitiy range. Ssb-band intensities of cytosolic and ribosomal fractions were then determined on the same exposure of a single immunoblot using AIDA Image Analyzer software (Raytest). The sum of the Ssb-band in the cytosolic fraction plus the Ssb-band in the ribosomal fraction (multiplied by the appropriate factor to adjust for loading) was set to 100% total. The fraction of ribosome-bound Ssb is given as a percentage of the total.

Supplementary References

1. Brachmann, C. B. *et al.* Designer deletion strains derived from Saccharomyces cerevisiae S288C: a useful set of strains and plasmids for PCR-mediated gene disruption and other applications. *Yeast* **14**, 115-132 (1998).
2. Heitman, J., Movva, N. R., Hiestand, P. C. & Hall, M. N. FK 506-binding protein proline rotamase is a target for the immunosuppressive agent FK 506 in Saccharomyces cerevisiae. *Proc. Natl Acad. Sci. USA* **88**, 1948-1952 (1991).
3. Rakwalska, M. & Rospert, S. The ribosome-bound chaperones RAC and Ssb1/2p are required for accurate translation in Saccharomyces cerevisiae. *Mol. Cell. Biol.* **24**, 9186-9197 (2004).
4. Gautschi, M. *et al.* RAC, a stable ribosome-associated complex in yeast formed by the DnaK-DnaJ homologs Ssz1p and zuotin. *Proc. Natl Acad. Sci. USA* **98**, 3762-3767 (2001).
5. Conz, C. *et al.* Functional characterization of the atypical Hsp70 subunit of yeast ribosome-associated complex. *J. Biol. Chem.* **282**, 33977-33984 (2007).
6. Gautschi, M., Mun, A., Ross, S. & Rospert, S. A functional chaperone triad on the yeast ribosome. *Proc. Natl Acad. Sci. USA* **99**, 4209-4214 (2002).
7. Jaiswal, H. *et al.* The chaperone network connected to human ribosome-associated complex. *Mol. Cell. Biol.* **31**, 1160-1173 (2011).
8. Ban, N. *et al.* A new system for naming ribosomal proteins. *Curr. Opin. Struct. Biol.* **24**, 165-169 (2014).
9. Gietz, R. D. & Sugino, A. New yeast-*Escherichia coli* shuttle vectors constructed with in vitro mutagenized yeast genes lacking six-base pair restriction sites. *Gene* **74**, 527-534 (1988).
10. Babiano, R. & de la Cruz, J. Ribosomal protein L35 is required for 27SB pre-rRNA processing in Saccharomyces cerevisiae. *Nucleic Acids Res.* **38**, 5177-5192 (2010).
11. Boeke, J. D., LaCroute, F. & Fink, G. R. A positive selection for mutants lacking orotidine-5k-phosphate decarboxylase activity in yeast: 5-fluoro-orotic acid resistance. *Mol. Gen. Genet.* **197**, 345-346 (1984).
12. Adams, P. D. *et al.* PHENIX: a comprehensive Python-based system for macromolecular structure solution. *Acta. Crystallogr. D Biol. Crystallogr.* **66**, 213-221 (2010).
13. Winn, M. D. *et al.* Overview of the CCP4 suite and current developments. Acta. Crystallogr. D Biol. Crystallogr. **67**, 235-242 (2011).
